# Structural requirements of KAI2 ligands for activation of signal transduction

**DOI:** 10.1073/pnas.2414779122

**Published:** 2025-02-20

**Authors:** Rito Kushihara, Akihiko Nakamura, Katsuki Takegami, Yoshiya Seto, Yusuke Kato, Hideo Dohra, Toshiyuki Ohnishi, Yasushi Todoroki, Jun Takeuchi

**Affiliations:** ^a^Department of Agriculture, Graduate School of Science and Technology, Shizuoka University, Shizuoka 422-8529, Japan; ^b^Department of Applied Life Sciences, Faculty of Agriculture, Shizuoka University, Shizuoka 422-8529, Japan; ^c^Research Institute of Green Science and Technology, Shizuoka University, Shizuoka 422-8529, Japan; ^d^Department of Life and Coordination-Complex Molecular Science, Institute for Molecular Science, National Institutes of Natural Sciences, Okazaki, Aichi 444-8787, Japan; ^e^Laboratory of Plant Chemical Regulation, Department of Agricultural Chemistry, School of Agriculture, Meiji University, Kanagawa 214-8571, Japan; ^f^Shizuoka Instrumental Analysis Center, Shizuoka University, Shizuoka 422-8529, Japan; ^g^Department of Biological Science, Graduate School of Integrated Science and Technology, Shizuoka University, Shizuoka 422-8529, Japan

**Keywords:** KAI2, receptor, ligand hydrolysis

## Abstract

Karrikin Insensitive 2 (KAI2), identified as the receptor protein for karrikins (KARs), which are smoke-derived seed germination stimulants, belongs to the same α/β-hydrolase family as D14, the receptor for strigolactones (SLs). KAI2 is believed to recognize an endogenous butenolide (KAI2 ligand; KL), but the identity of this compound remains unknown. Recent studies have suggested that ligand hydrolysis by KAI2 is a prerequisite for receptor activation to induce interaction with the target proteins, similar to the situation with D14. However, direct experimental evidence has been lacking. Here, we designed KAI2 ligands (carba-dMGers) whose butenolide rings were modified so that they cannot be hydrolyzed or dissociated from the original ligand molecule by KAI2, by structurally modifying dMGer, a potent and selective KAI2 agonist. Using these dMGer analogs, we found that the strongly bioactive ligand, (+)-dMGer, was hydrolyzed by KAI2 at a lower enzymatic rate compared with the weakly bioactive ligand, (+)-1′-carba-dMGer, and the hydrolyzed butenolide ring of (+)-dMGer was transiently trapped in the catalytic pocket of KAI2. Additionally, structural analysis revealed that (+)-6′-carba-dMGer bound to the catalytic pocket of KAI2 in the unhydrolyzed state. However, this binding did not induce the interaction between KAI2 and SMAX1, indicating that ligand binding to the receptor alone was not sufficient for KAI2 signaling. This study showed experimental data from a ligand structure–activity study that ligand hydrolysis and subsequent covalent adduct formation with the catalytic triad plays a key role in KAI2 activation, providing insight into the chemical structure of the *Arabidopsis* KL.

## Significance

Karrikin Insensitive 2 (KAI2) proteins are plant α/β-hydrolases with dual receptor-enzyme function that perceive smoke-derived karrikins (KARs) and an endogenous, yet undiscovered, KAI2 ligand (KL). Upon ligand binding, KAI2 is activated and interacts with its target proteins, including MAX2 and SMAX1, which initiate the physiological responses. Activation of KAI2 is thought to require hydrolysis of the ligand; however, this has not been experimentally proven. Here, we developed KAI2 ligands (carba-dMGers) that resist hydrolysis by KAI2, by structural modification of dMGer, a potent KAI2 agonist. These chemical tools provided experimental evidence that the ligand cleavage reaction (hydrolysis and dissociation) is crucial for the induction of KAI2 signaling. Our research also suggested that the ligand interaction with the catalytic serine was not sufficient for KAI2 activation.

In the past decade, many phytohormone agonists and antagonists that bind to and activate or inhibit receptors have been developed as chemical tools for chemical biology and chemical genetics studies (1–4). The application of these tools has allowed instantaneous, reversible, and conditional modification of a phenotype, thereby circumventing the limitations of classical genetic approaches, including genetic redundancy, lethality, and pleiotropy (5). Thus, receptor agonists and antagonists have contributed considerably to understanding the mode of action of phytohormones. For example, structure–activity relationship studies have allowed the determination of the structural requirements essential for the bioactivity of the agonists/antagonists. However, only a few compounds have been successfully used to elucidate biological phenomena among the numerous chemical tools developed to date. This observation suggests that it is important not only to develop chemical tools but also to demonstrate their usefulness (e.g., know how to use the tools).

Karrikin Insensitive 2 (KAI2) is a member of the α/β-hydrolase family that was first identified as a receptor protein of karrikins (KARs) (6, 7). KARs are a class of butenolide compounds produced by wildfires, which are found in the smoke released from the combustion of plant material and act as seed germination inducers for fire-prone species (8, 9). Subsequent studies have revealed that KAR responses are not limited to fire-prone species and regulate several physiological processes, such as germination, seed photomorphogenesis, and root growth, in many plant species (10–13). However, there is currently no evidence that KARs are produced by living plants. Therefore, KARs are thought to be strictly abiotic, or at least not endogenous, signal molecules. However, several biological experiments have suggested that KARs mimic an endogenous KAI2 ligand (KL) (14, 15). For example, a *kai2* mutant showed seed and seedling phenotypes that were opposite to those of the wild-type plants treated with KARs (6, 16), implying that the responses to KL signaling had been lost in the *kai2* mutant. In addition, aqueous phase extracts from *Arabidopsis* leaves stimulated the expression of a KAR-responsive gene in a KAI2-dependent manner (17). Most recently, the volatile organic compound (VOC) (−)-germacrene D, a monocyclic sesquiterpene without a lactone ring, has been reported to be an endogenous ligand for a petunia KAI2 homolog (PhKAI2ia) (18). PhKAI2ia senses (−)-germacrene D and activates VOC signaling though the degradation of PhSMAX1a [collectively orthologous to SUPPRESSOR-OF-MAX2-1 (SMAX1) in *Arabidopsis*] in petunia pistils. However, there is currently no experimental evidence that (−)-germacrene D activates KAI2 receptors in other plant species, and we have observed that (−)-germacrene D does not act as a KAI2 agonist in *Arabidopsis* (*SI Appendix*, Fig. S1*A*).

While research efforts toward the identification of KL are still currently underway, the outline of the physiological response mechanism mediated by KAI2 has been clarified. KAI2 is an ancient paralog of the strigolactone receptor DWARF14 (D14)/DECREASED APICAL DOMINANCE2 (DAD2)/RAMOSUS3 (RMS3) (19–21). SLs, a class of carotenoid-derived plant hormones, and KARs share a common substructure, a butenolide ring, which is essential for bioactivity, and their signaling mechanisms are quite similar. D14 and KAI2 in *Arabidopsis* bind and regulate the activity of the F-box protein MORE AXILLARY BRANCHES2 (MAX2, known as DWARF3 in rice). MAX2 acts as an Skp1, cullin, and F-box (SCF)-type E3 ligase complex to recruit members of the SMAX1 and SMAX1-LIKE (SMXL) family for degradation via the proteasome-ubiquitin pathway (21–23). MAX2 targets specific SMXL proteins as substrates through D14 and KAI2. Activation of D14 by SLs induces degradation of SMXL6, SMXL7, and SMXL8 in *Arabidopsis* (DWARF53 in rice), thereby allowing SL signaling to proceed (24–29). In contrast, KAI2 promotes the degradation of SMAX1 and SMXL2 in *Arabidopsis* (OsSMAX1 in rice) to induce KAR-associated traits, including seed germination and seedling photomorphogenesis (29–32). However, in some cases (e.g., hypocotyl elongation), the division of roles between KAI2 and D14 may not be so clear. Recent studies have indicated that D14 can bind to SMAX1 and SMXL2 in the presence of SLs and induce the degradation of SMAX1/SMXL2 (29, 33).

KAI2 and D14 are α/β-hydrolases containing a Ser-His-Asp catalytic triad in their hydrophobic substrate-binding pockets, and therefore both proteins display hydrolytic activity toward various SL analogs, including GR24, desmethyl-GR24, and debranones compounds (34–36). In SL signaling, the butenolide group (D-ring) of SLs is hydrolyzed by D14 and forms a transient covalent bond with the catalytic histidine (20, 21), and consequently SLs induce a conformational change or/and conformational destabilization of D14, facilitating the interaction of D14 with SMXL6/7/8 and MAX2 (24, 25, 27, 37). Although the precise role of this ligand hydrolysis remains controversial (21, 23, 38), it appears to be required for SL signaling to be fully activated (39). For example, SL hydrolysis may be the driving force in releasing polyubiquitinated SMXLs from the D14–MAX2–SMXL complex and inducing proteasomal degradation of the SMXLs (40). KAI2 signaling proceeds similarly, and thus it is assumed that ligand hydrolysis by KAI2 is essentially the same as for D14 (15). However, in KAI2, two types of agonists have been reported (one type is not hydrolyzed by KAI2, e.g., the KARs, and the other can be hydrolyzed, e.g., GR24*^ent^*^-5DS^), which is one of the reasons why the identification of KL has been difficult. Recent studies have reported that *Arabidopsis* KAI2 (AtKAI2) and several homologs from moss (PpKAI2-like) and pea (PsKAI2B) hydrolyze GR24*^ent^*^-5DS^ and form covalent adducts with a mass of 96 Da (41–43). In addition, several observations have suggested that KARs do not directly activate KAI2 and that KARs must be metabolized in vivo before they can be recognized by KAI2 (39, 44). Therefore, the hydrolyzable butenolide ring, which covalently modifies a catalytic triad residue, may be a common structural feature of KAI2 agonists that exhibit KAR-like biological activity. However, there has been no direct experimental evidence that ligand hydrolysis and the formation of a covalent adduct with a catalytic residue are essential for KAI2 signal transduction.

Here, we designed chemical tools that can bind to KAI2 but are not hydrolyzed and/or are unable to form covalent adducts to elucidate the role of ligand hydrolysis in KAI2 signaling. dMGer (45), which has been reported to be a potent and selective KAI2 agonist, was used as a lead compound to create carba-dMGer compounds. Herein, we describe the design, synthesis, and in vitro and in vivo properties of these carba-dMGer compounds and discuss the precise significance of the ligand hydrolysis and subsequent covalent bond modification for KAI2 signaling.

## Results and Discussion

### Design and Synthesis.

**Fig. 1.**
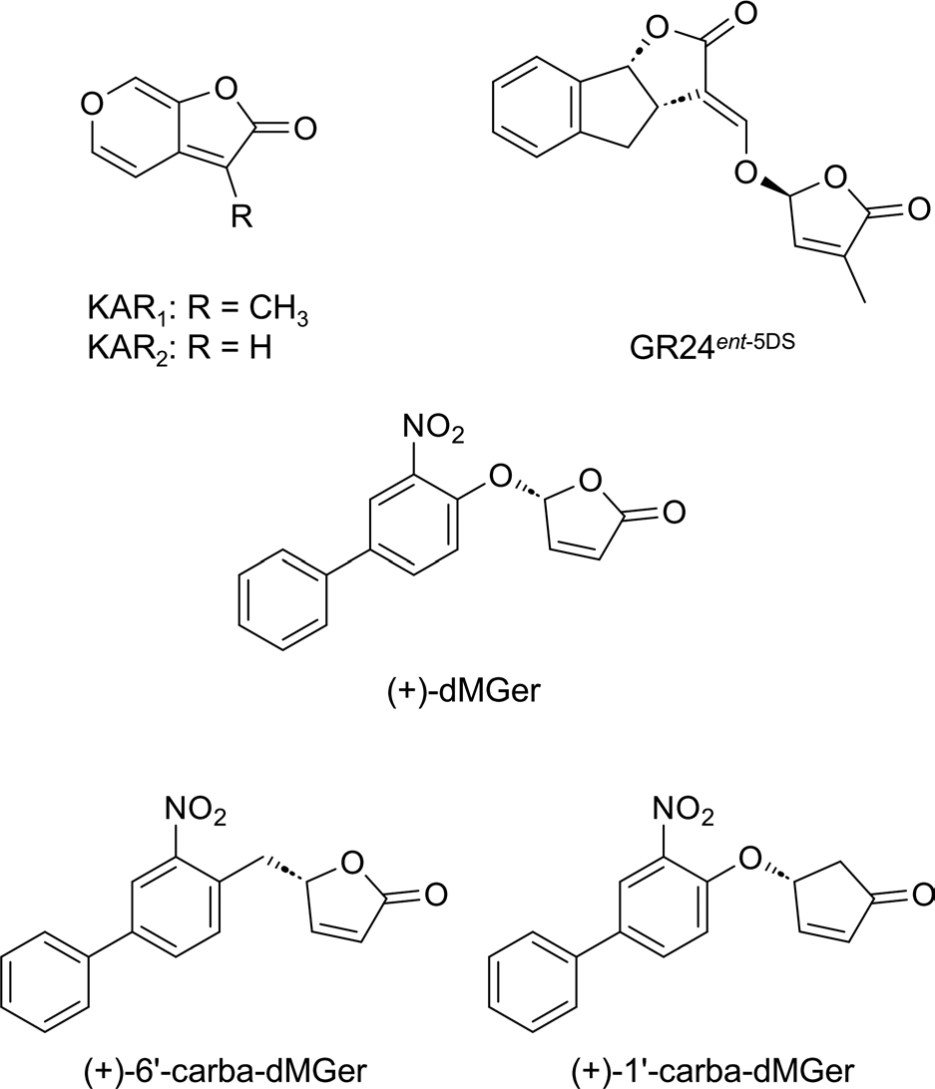
Structures of KAI2 agonists and hydrolysis-resistant (+)-carba-dMGer analogs.

The most widely used KAI2 agonist is GR24*^ent^*^-5DS^, a 2′*S*-configured synthetic SL analog, and therefore, it would appear ideal to design nonhydrolyzable KAI2 ligands by derivatization of GR24*^ent^*^-5DS^. However, GR24-type nonhydrolyzable analogs (e.g., 1′-carba-GR24 and 6′-carba-GR24) have three chiral carbons (C-3a, -8b, and -2′) in the molecule, and thus, four stereoisomers are produced during chemical synthesis. These stereoisomers are routinely separated using time-consuming HPLC prior to biological applications. Moreover, the synthesis of 6′-carba-GR24 requires more than 10 steps, which makes it difficult to use as a chemical tool. Therefore, we selected a desmethyl germinone (dMGer; Fig. 1), which has a single chiral carbon and can be easily synthesized in two steps (45), as the core for constructing nonhydrolyzable KAI2 ligands. dMGer is a more potent and selective KAI2 agonist than GR24*^ent^*^-5DS^, suggesting that dMGer is a suitable lead compound. Substrate recognition by KAI2 is thought to be triggered by nucleophilic attack on the butenolide carbonyl group by the catalytic serine of KAI2, which opens up the butenolide ring (43). Therefore, we designed 1′-carba-dMGer, in which the butenolide ring of dMGer was replaced with a cyclopentenone ring, as a nonhydrolyzable KAI2 ligand (Fig. 1). 6′-Carba-dMGer, in which the phenol ether oxygen of dMGer was replaced by a CH_2_ group, was also designed as a chemical tool that cannot form a covalent adduct with KAI2 because the butenolide ring does not dissociate from the biphenyl moiety. The carba-dMGer compounds were synthesized as shown in *SI Appendix*, Fig. S2. 4-Hydroxy-3-nitrobiphenyl was introduced to 4-hydroxycyclopent-2-en-1-one (compound **1**) (46) via the Mitsunobu reaction to generate (±)-1′-carba-dMGer. (±)-6′-Carba-dMGer was synthesized from 2-(3-nitro-[1,1′-biphenyl]-4-yl)ethan-1-ol (compound **2**) (47). The oxidation with Dess-Martin periodinane resulted in aldehyde **3**, which was treated with vinylmagnesium bromide to yield alcohol **4**. The introduction of an acrylaldehyde by treating alcohol **4** with acryloyl chloride in the presence of triethylamine and 4-(dimethylamino) pyridine yielded allylic ester **5** (48). The allylic ester **5** was then treated with 6 mol% second generation Grubbs catalyst to generate (±)-6′-carba-dMGer. Before investigating the effects in vitro and in vivo, both these synthesized compounds and (±)-dMGer were optically resolved by HPLC on a chiral column.

To determine the absolute stereochemistry of the (+)- and (−)-6′-carba-dMGer enantiomers, optically pure (+)-6′-carba-dMGer was prepared from optically pure (+)-compound **4**, which was obtained by optical resolution of the racemate using semipreparative HPLC with a chiral column. The absolute stereochemistry of both enantiomers of compound **4** was determined by an advanced Mosher’s method using the ^1^H NMR spectra of the *R*-α-methoxy-α-(trifluoromethyl)phenyl acetates (MTPAs) (49). The *R*-MTPA esters were prepared with *S*-MTPA-Cl in pyridine (*SI Appendix*, Fig. S3). The geometries, which were optimized with B3LYP/6-31G(d) from the initial geometries with the ideal MTPA plane, retained the plane sufficiently to apply the advanced Mosher’s method (*SI Appendix*, Fig. S3). The phenyl moiety in MTPA was positioned to have a shielding effect on the vinyl group of *S*-**4** and the nitrophenyl moiety of *R*-**4**. ^1^H NMR analysis showed that the vinyl protons of the (+)-isomer and the nitrophenyl protons of the (−)-isomer were shielded. Thus, the absolute configurations of (+)-**4** and (−)-**4** were determined to be *S* and *R*, respectively. Optically pure *S*-(+)-6′-carba-dMGer was synthesized from the optically pure *S*-(+)-**4** in the same manner as described above (*SI Appendix*, Fig. S4). The absolute configurations of (+)-/(−)-dMGer and (+)-/(−)-1′-carba-dMGer were assigned on the basis of the circular dichroism (CD) spectra with reference to *S*-(+)-/*R*-(−)-6′-carba-dMGer (*SI Appendix*, Fig. S5). According to the CD spectrum of the respective enantiomers, the absolute configuration of C-2′ was determined to be *R* for (+)-dMGer/(+)-1′-carba-dMGer and *S* for the (−)-isomers. Structural superimposition of *R*-(+)-dMGer and *R*-(+)-1′-carba-dMGer with *S*-(+)-6′-carba-dMGer indicated that there was a high similarity between the core structures (the C/O-butenolide rings) in the three compounds.

### Biological and Physiological Effects of the dMGer Analogs.

**Fig. 2.**
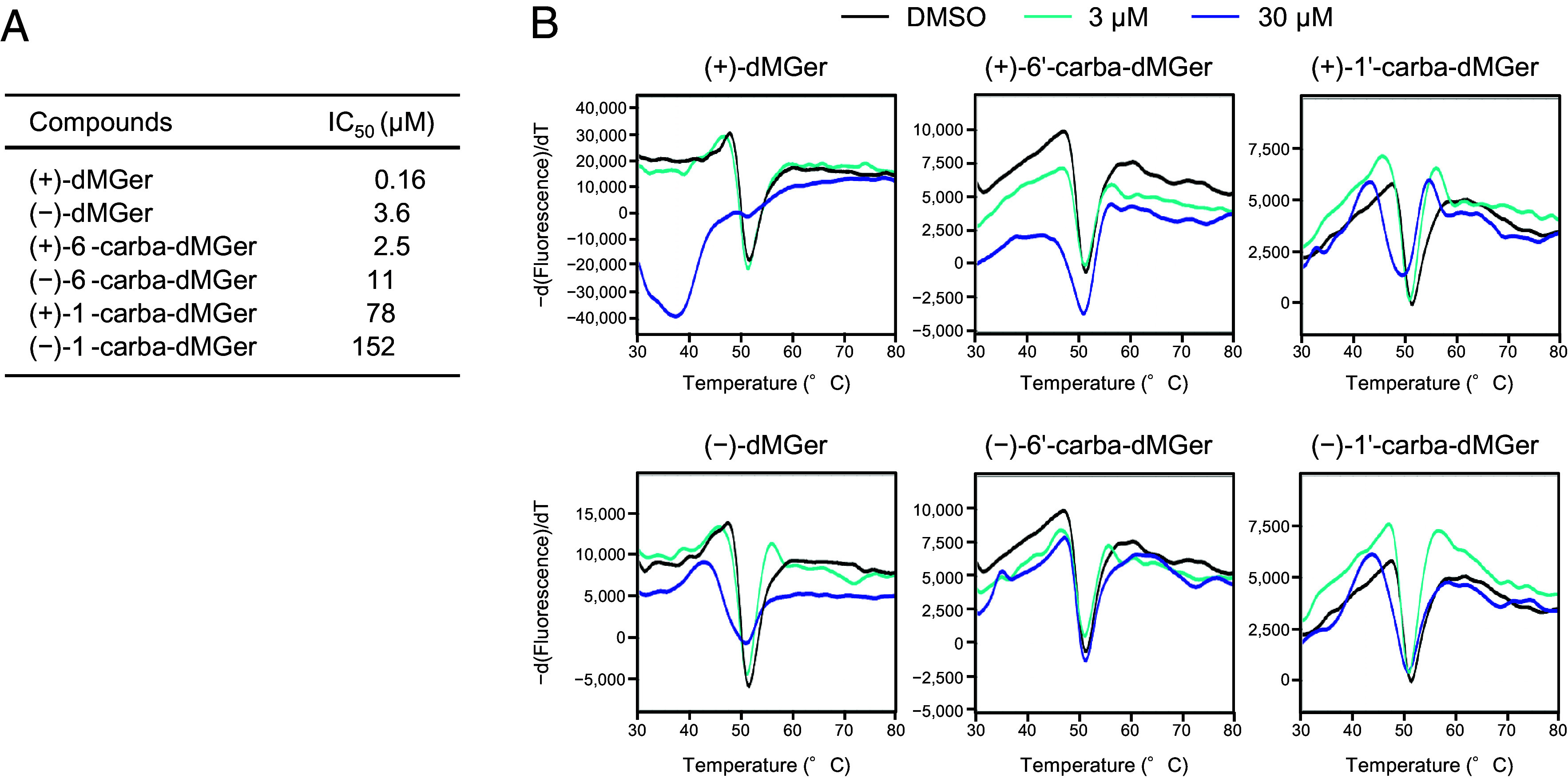
Evaluation of the binding activity of dMGer analogs toward KAI2. (*A*) Inhibitory effects of dMGer analogs on KAI2 hydrolytic activity. Relative fluorescence of the dYLG probe in the presence of purified KAI2 and increasing concentrations of the test compounds. The listed IC_50_ values are the averages from three experiments. (*B*) Thermal shift response assays of KAI2 in response to (+)-/(−)-dMGer, (+)-/(−)-6′-carba-dMGer, and (+)-/(−)-1′-carba-dMGer. The melting temperature curves of KAI2 after incubation with the indicated concentration of each chemical are shown.

We first conducted competitive inhibition assays to determine whether the carba-dMGers could bind to the ligand binding pocket of KAI2. In these assays, a profluorescent probe, desmethyl-Yoshimulactone green (dYLG), was used as a substrate, and the KAI2 binding activity was assessed by the inhibitor concentrations necessary to halve the response (IC_50_) for fluorophore formation. All the (+)-dMGer analogs showed a stronger inhibitory activity than the (−)-isomers, and the butenolide compounds (dMGer and 6′-carba-dMGer) were more potent inhibitors than 1′-carba-dMGer, with a cyclopentenone ring instead of the butenolide ring (Fig. 2*A* and *SI Appendix*, Fig. S6). The IC_50_ values of (+)-dMGer, (+)-6′-carba-dMGer, and (+)-1′-carba-dMGer were 0.16, 2.5, and 78 µM, respectively, when 0.5 µM dYLG was used as the substrate. Differential scanning fluorimetry (DSF) assays were also performed to evaluate the effect of the dMGer analogs on KAI2 destabilization. In the DSF assay, ligand–protein interactions are monitored by changes in the melting temperature (*T*_m_); effective agonists, such as GR24*^ent^*^-5DS^ and dGR24*^ent^*^-5DS^, lower the *T*_m_ value, that is, thermally destabilize the KAI2 protein (15, 35). (+)-dMGer dramatically decreased the *T*_m_ value of KAI2 from 51.6 to 33.4 °C with increasing ligand concentrations. (−)-dMGer, (+)-/(−)-6′-carba-dMGer, and (+)-/(−)-1′-carba-dMGer also caused a *T*_m_ shift of KAI2, but the effect was less than that of (+)-dMGer (Fig. 2*B*). These results indicated that 6′-carba-dMGer and 1′-carba-dMGer bind to the ligand-binding pocket of KAI2.

**Fig. 3.**
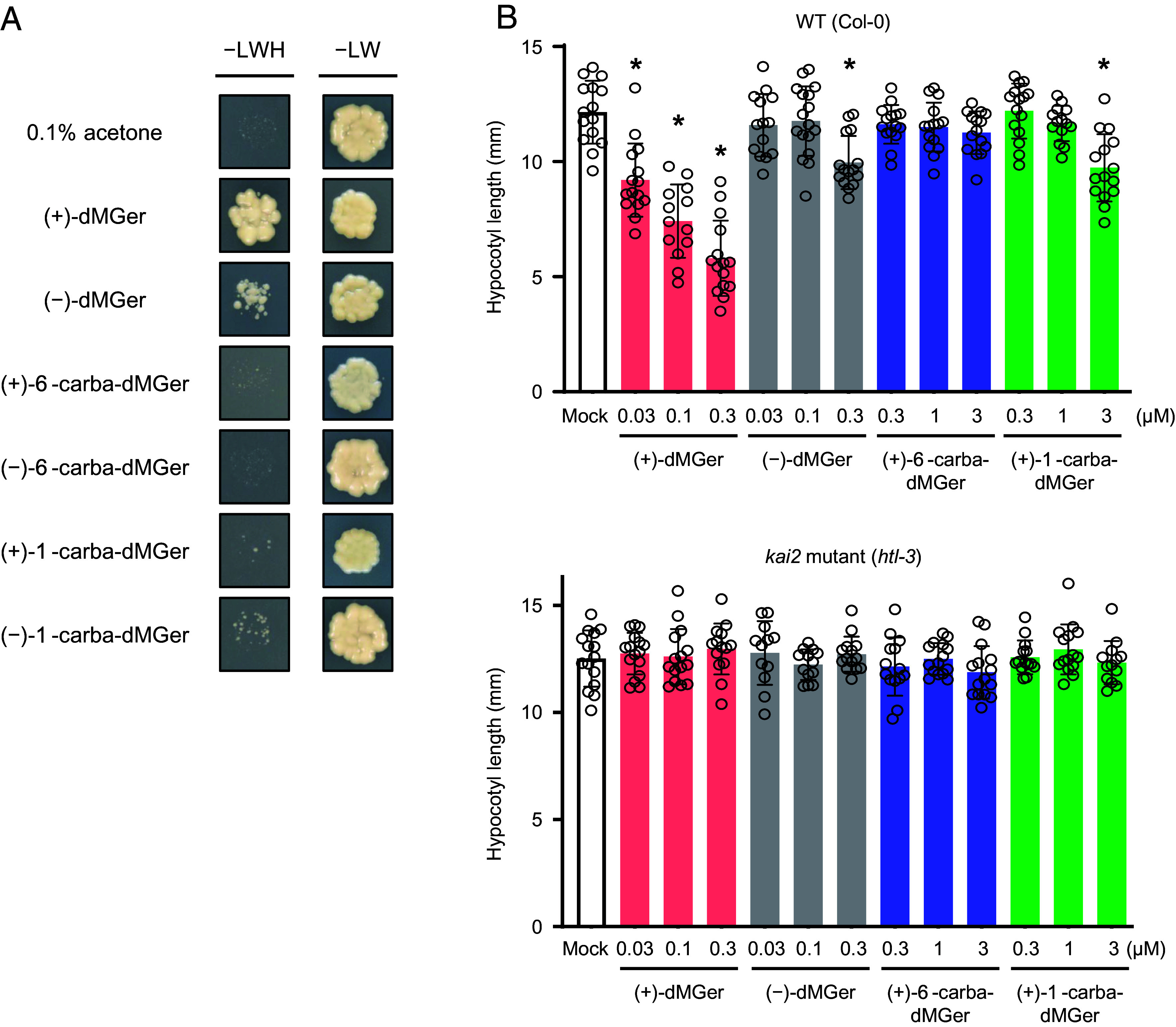
Effects of 6′-carba-dMGer and 1′-carba-dMGer compared with those of (+)-dMGer. (*A*) Analysis of the KAI2–SMAX1 interaction in the presence of (+)-/(−)-dMGer, (+)-/(−)-6′-carba-dMGer, or (+)-/(−)-1′-carba-dMGer using the yeast two-hybrid method. Yeast transformants were spotted onto the selective medium [SD−Leu/−Trp/−His (−LWH)] in the absence or presence of 1 µM dMGer analogs. (*B*) *Arabidopsis* (Col-0) hypocotyl elongation response to (+)-dMGer, (−)-dMGer, (+)-6′-carba-dMGer, and (+)-1′-carba-dMGer in WT and *kai2* mutant (*htl-3*). Data are the means ± SD (*n* = 13 to 15). Asterisks indicate a significant difference compared with mock-treated seedlings: *P < 0.05 (Dunnett’s test). Small circles indicate each data point. Similar results were obtained in three independent experiments.

Next, to evaluate whether the KAI2 binding ability of (+)-/(−)-dMGer, (+)-/(−)-6′-carba-dMGer, and (+)-/(−)-1′-carba-dMGer was physiologically relevant, we performed yeast two-hybrid (Y2H) assays and in vivo assays. In the Y2H assays, we used SMAX1 as a partner protein of KAI2 (45) because the ligand-induced interaction between KAI2 and MAX2 have been considered difficult to assess in this system (50). (+)-dMGer induced the interaction of KAI2 with SMAX1 at a 1 µM concentration, and (−)-dMGer showed a slightly weaker response than the (+) enantiomer (Fig. 3*A*). In contrast, 6′-carba-dMGer and 1′-carba-dMGer did not induce the KAI2–SMAX1 interaction. At 10 µM; however, the dMGer analogs except for 6′-carba-dMGer inhibited yeast growth (*SI Appendix*, Fig. S7). In the *Arabidopsis* hypocotyl elongation assay, in which KAI2 agonists exhibit inhibitory activity, (+)-dMGer strongly inhibited hypocotyl elongation at a concentration range of 0.03 to 0.3 µM (Fig. 3*B*). (−)-dMGer and (+)-1′-carba-dMGer showed a weaker inhibitory effect than that of (+)-dMGer, and (−)-1′-carba-dMGer and (+)-/(−)-6′-carba-dMGer did not inhibit hypocotyl elongation at a concentration range of 0.3 to 3 µM (Fig. 3*B* and *SI Appendix*, Fig. S8). The weak inhibitory effect of (+)-1′-carba-dMGer at 3 µM in wild-type *Arabidopsis* was not observed for the *kai2* knockout mutant (Fig. 3*B* and *SI Appendix*, Fig. S8). This result suggested that (+)-1′-carba-dMGer weakly activated the KAI2 protein, although it did not induce activity caused by the KAI2–SMAX1 interaction under the Y2H conditions used in the present study. The effect of (+)-/(−)-dMGer, (+)-6′-carba-dMGer, and (+)-1′-carba-dMGer on the thermoinhibition of *Arabidopsis* seeds was also investigated. (+)-dMGer promoted the germination of thermoinhibited seeds from a concentration of 0.01 µM (*SI Appendix*, Fig. S9), as expected of a KAI2 agonist. (−)-dMGer and (+)-1′-carba-dMGer slightly alleviated thermoinhibition at a concentration of 10 µM, but (+)-6′-carba-dMGer showed no effect on the thermoinhibition (*SI Appendix*, Fig. S9), which was consistent with the results of the hypocotyl elongation assay. These physiological data indicated that (+)-1′-carba-dMGer acted as a weak agonist of KAI2, while (+)-6′-carba-dMGer did not act as an agonist. It was also found that KAI2 clearly recognized the C2′ configuration of the butenolide ring in dMGer, i.e., KAI2 had a strong preference for 2′*R*-configured (+)-dMGer over 2′*S*-configured (−)-dMGer (Figs. 2 and 3), which was opposite to the previously observed preference for GR24*^ent^*^-5DS^ over GR24^5DS^ (51). These data, together with the results of the in vitro assays, suggested that ligand binding to the receptor alone was not sufficient for KAI2 signaling and that ligand hydrolysis and subsequent covalent adduct formation play a key role in the full activation of KAI2. These observations are similar to the activation mechanism of ShHTL7, an SL receptor of *Striga hermonthica*, where the potencies of nonhydrolyzable carba-H-SPL7 and 1′-carba-SPL7 are much lower than that of H-SPL7, a hydrolyzable agonist (52).

### Biochemical Characterization of the Effect of the dMGer Analogs on KAI2 Activation.

**Fig. 4.**
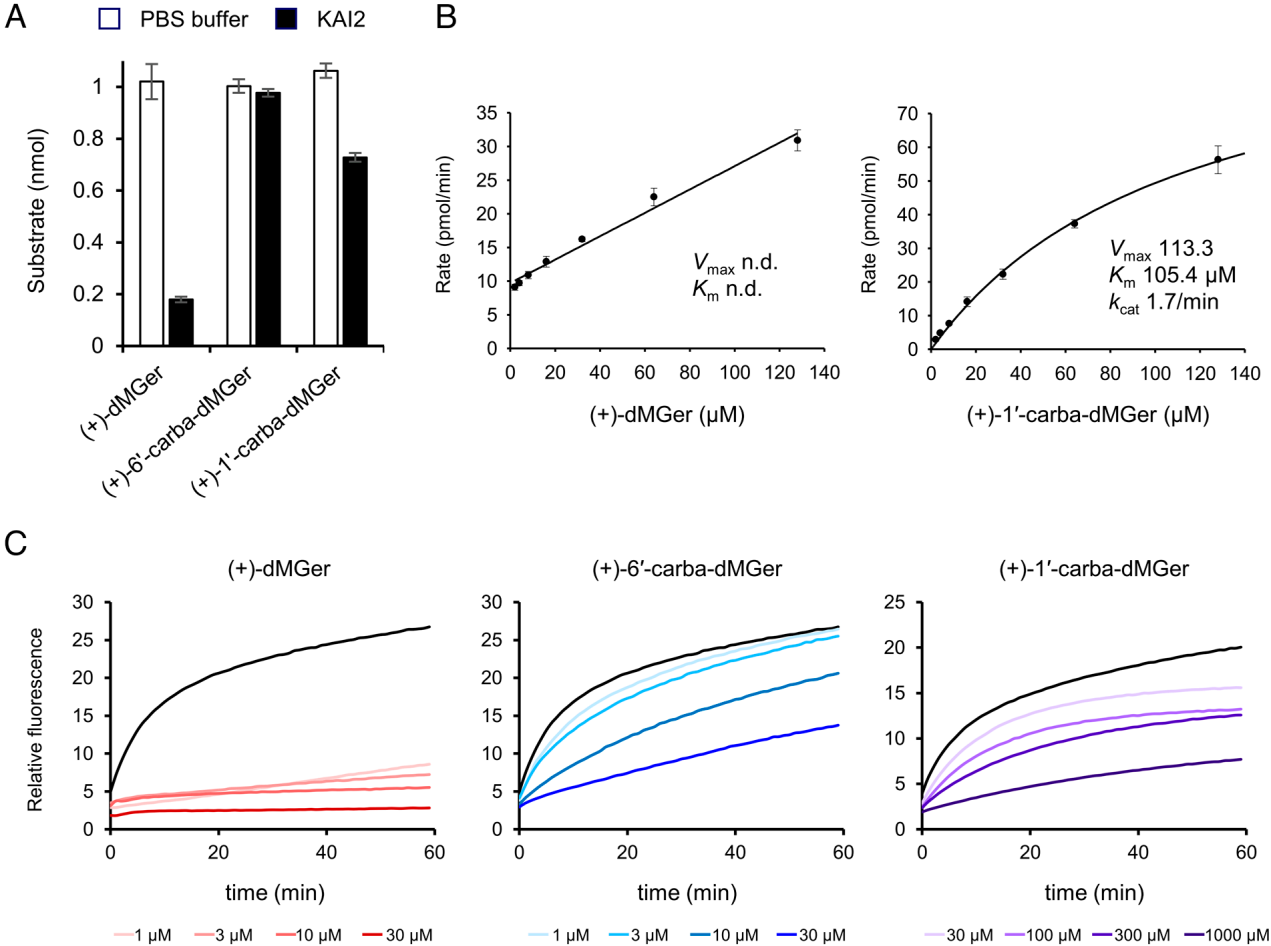
Enzymatic kinetics revealed that the metabolic velocity of (+)-dMGer by KAI2 was slow. (*A*) Enzymatic degradation of (+)-dMGer, (+)-6′-carba-dMGer, and (+)-1′-carba-dMGer by KAI2. Each test compound (10 µM) was incubated with or without KAI2 (3 µM) (*n* = 3; error bars represent the SDs.). (*B*) KAI2 steady-state kinetics reaction velocity with (+)-dMGer (*Left*) or (+)-1′-carba-dMGer (*Right*). Enzymatic activity was defined as the amount of 4-hydroxy-3-nitrobiphenyl (leaving group) produced over 10 min in the presence of 0.63 µM KAI2 protein and 2, 4, 8, 16, 32, 64, or 128 µM (+)-dMGer or (+)-1′-carba-dMGer. Error bars represent the SDs of three replicates. (*C*) Chemical inhibition of KAI2-catalyzed hydrolysis by the indicated concentrations of (+)-dMGer, (+)-6′-carba-dMGer (1, 3, 10, and 30 µM), and (+)-1′-carba-dMGer (30, 100, 300, and 1,000 µM). Fluorescence intensity progress curves during dYLG hydrolysis, monitored (*λ*_max_ 520 nm) at 22 °C. Fluorescence intensity was normalized to a blank (without KAI2) value of 1 and is expressed as the relative activity. Black traces indicate 0 µM (+)-dMGer analogs (no inhibitors).

(+)-dMGer and (+)-1′-carba-dMGer showed, respectively, strong and weak KAI2 agonist activity but (+)-6′-carba-dMGer did not act as an agonist in the physiological assays. Therefore, the hydrolyzability of these compounds and their respective enantiomers by KAI2 was evaluated based on the decrease in the amounts of the substrates after incubation with KAI2 (Fig. 4*A* and *SI Appendix*, Fig. S10). (+)-dMGer was approximately 80% cleaved in 10 min, but (+)-6′-carba-dMGer was not hydrolyzed, consistent with predictions based on the molecular design of the compounds. Unexpectedly, (+)-1′-carba-dMGer was approximately 30% cleaved by KAI2, although it has a nonhydrolyzable cyclopentenone ring, instead of the butenolide ring. (−)-dMGer analogs exhibited similar ligand profile to that of the (+)-enantiomers (*SI Appendix*, Fig. S10), suggesting that KAI2 hydrolyzes dMGer regardless of the C2′ configuration. The cleavage of (+)-1′-carba-dMGer probably occurred via nucleophilic attack on the C2′ atom of the cyclopentenone ring by the NE2 atom of His246 of KAI2 (53) (*SI Appendix*, Fig. S11). This result might explain the weak agonistic activity of (+)-1′-carba-dMGer in plants. To assess whether the ligand hydrolyzability was related to the biological activity, we analyzed the enzymatic kinetics of KAI2 toward (+)-dMGer and (+)-1′-carba-dMGer. The hydrolyzability was determined by measuring the release of 4-hydroxy-3-nitrobiphenyl after hydrolysis of (+)-dMGer and (+)-1′-carba-dMGer by the KAI2 protein. We found that the hydrolytic activity of KAI2 toward (+)-dMGer was strong but were unable to determine any kinetic parameters because the activity was already saturated at the initial concentration (2 µM) in the steady state (Fig. 4*B*). Therefore, we defined *k*_cat_ as the rate constant of the pre-steady-state phase and *K*_1/2_ as the (+)-dMGer concentration at half maximal velocity (*V*_max_), in accordance with previous studies on SL receptors (20) (*SI Appendix*, Fig. S12). In contrast, KAI2 showed saturable hydrolytic activity toward (+)-1′-carba-dMGer also in the steady state (Fig. 4*B* and *SI Appendix*, Fig. S12). (+)-dMGer (*K*_1/2_ = 4.3 µM) exhibited higher affinity for KAI2 than (+)-1′-carba-dMGer (*K*_1/2_ = 69.7 µM), while the catalytic constant was lower than that of (+)-1′-carba-dMGer [*k*_cat_ = 0.20 and 0.83 min^−1^ for (+)-dMGer and (+)-1′-carba-dMGer, respectively].

Next, we analyzed the enzymatic properties of the (+)-dMGer hydrolysis by KAI2 by investigating two successive additions of (+)-dMGer. After completion of the hydrolysis of (+)-dMGer (3 µM) by KAI2 protein (3 µM), (+)-dMGer (3 µM) was successively added to the reaction mixture and the amount of 4-hydroxy-3-nitrobiphenyl was quantified. The (+)-dMGer added later was also hydrolyzed by KAI2, but the reaction was incomplete (approximately 40% hydrolysis) (*SI Appendix*, Fig. S12). This result suggested that KAI2 was inactivated, although not completely, after the hydrolysis of a (+)-dMGer molecule. In addition, dYLG assays were performed to evaluate the persistence of the KAI2 inhibitory effect by (+)-dMGer analogs (Fig. 4*C*). (+)-dMGer almost completely blocked the hydrolysis of dYLG for 60 min, while treatment with (+)-1′-carba-dMGer or (+)-6′-carba-dMGer resulted in an increase in the fluorescence intensity over time. These results suggested that the product of the (+)-dMGer hydrolysis reaction, probably the butenolide ring moiety, formed a covalent intermediate with KAI2, with the release of 4-hydroxy-3-nitrobiphenyl observed in our enzyme assay (*SI Appendix*, Fig. S12), but the hydrolysis reaction of (+)-1′-carba-dMGer did not form such an intermediate. The formation of this intermediate may explain the lower *k*_cat_ value of (+)-dMGer compared with that of (+)-1′-carba-dMGer.

**Fig. 5.**
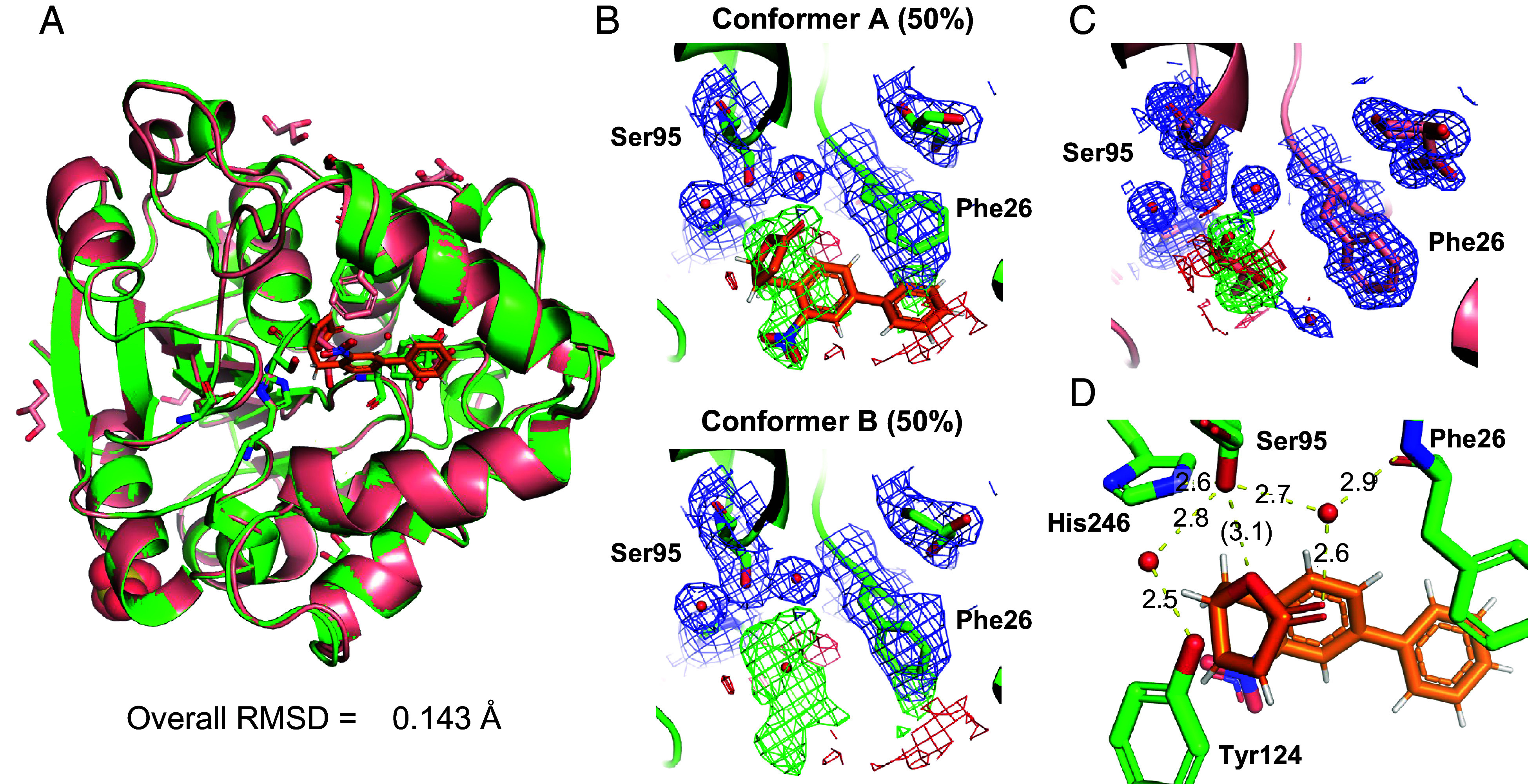
Crystal structures of KAI2 with and without (+)-6′-carba-dMGer. (*A*) Superimposition of the overall structures of KAI2-(+)-6′-carba-dMGer (green) and *apo*-KAI2 (pink). The overall RMSD was 0.143 Å. (*B*) Fo-Fc omit map for (+)-6′-carba-dMGer and water molecule binding at the catalytic site. KAI2 formed conformer A, in which (+)-6′-carba-dMGer was bound, and conformer B without the ligand. (*C*) Fo-Fc omit map for glycerol in *apo*-KAI2. (*D*) Distances between atoms that possibly form hydrogen bonds with (+)-6′-carba-dMGer in the catalytic site of KAI2.

To elucidate the molecular interaction of KAI2 with the (+)-dMGer analogs, we analyzed the crystal structure of the KAI2–(+)-6′-carba-dMGer complex. We examined only the KAI2–(+)-6′-carba-dMGer complex because we were unable to crystallize the KAI2–(+)-dMGer and KAI2–(+)-1′-carba-dMGer complexes. The complex of recombinant KAI2 bound to (+)-6′-carba-dMGer was determined at 2.1 Å resolution [Protein Data Bank (PDB) ID: 8ZVN; the refinement and structure statistics are summarized in *SI Appendix*, Table S1]. A structural comparison of the backbone atoms between *apo*-KAI2 and KAI2-(+)-6′-carba-dMGer did not reveal any appreciable differences (Fig. 5*A*), and the RMSD was 0.143 Å for the main-chain Cα atoms. The electron density map indicated that the KAI2-(+)-6′-carba-dMGer crystal structure was composed of two conformers: The ligand was bound in conformer A, whereas the ligand was not observed in conformer B (Fig. 5*B*). In conformer A, the butenolide ring of (+)-6′-carba-dMGer faced toward the bottom of the catalytic pocket, while the nitrobiphenyl moiety was oriented toward the access groove of the pocket and was partially exposed to the solvent. Additionally, the orientation of the side-chain phenyl ring of Phe26 in KAI2 was shifted in conformer A compared with *apo*-KAI2 and conformer B (Fig. 5*C*). The phenyl ring of Phe26 probably formed a π–π interaction with the phenyl ring of (+)-6′-carba-dMGer in conformer A. In conformer A, the butenolide ring of (+)-6′-carba-dMGer formed a strong hydrogen bond network with Phe26, Ser95, Tyr124, and His246 that was mediated by two water molecules (Fig. 5*D*). However, the *O*-1′ atom, not the 5′-carbonyl oxygen, was oriented toward the catalytic serine, probably because of the methylene group introduced instead of the phenol ether oxygen of (+)-dMGer. This orientation of the butenolide ring may prevent nucleophilic attack from the serine and explain the nonhydrolyzability of (+)-6′-carba-dMGer (Fig. 4*A*).

In the decade since the discovery of KAI2 as a receptor for KARs, the signaling components have been identified from KAR-insensitive mutants. Similar to the SL pathway, the ligand-dependent degradation of negative regulators, such as SMAX1 and SMXL2, triggers KAI2 signaling. Degradation of SMAX1/SMXL2 occurs in a manner that requires the F-box protein MAX2 and the α/β-hydrolase receptor KAI2, which contains a conserved catalytic Ser-His-Asp triad and hydrolyzes agonistic ligands, such as GR24*^ent^*^-5DS^. Thus, whether ligand hydrolysis is essential for KAI2 signaling is an important question regarding the signaling mechanism of KAI2. Because mutation of the catalytic serine (Ser95) of KAI2 abolished hydrolytic activity and KAR responses (15), the ligand hydrolysis or the ligand interaction with catalytic residues is thought to be a prerequisite for KAI2 signaling. However, direct experimental evidence of the effect of the ligand has been lacking. Therefore, in the present study, we synthesized structural analogs of dMGer, a potent KAI2 agonist (45), as chemical tools to investigate the role of ligand hydrolysis in KAI2 signaling. These carba-dMGer analogs were designed to be unable to be hydrolyzed by KAI2 or unable to form covalent adducts with the catalytic residues of KAI2. The biological and enzymatic data revealed that the (+)-carba-dMGer analogs bound to the ligand-binding pocket of KAI2, similar to (+)-dMGer. In addition, analysis of the crystal structure of KAI2–(+)-6′-carba-dMGer indicated that (+)-6′-carba-dMGer bound to the catalytic pocket of KAI2 in an intact state. However, this interaction between (+)-6′-carba-dMGer and KAI2 did not activate the receptor in vivo, implying that the formation of a covalent adduct between a ligand hydrolyzate and the catalytic triad plays a key role in inducing the interactions between KAI2 and its target proteins, including SMAX1 and MAX2.

To evaluate this hypothesis, we incubated (+)-dMGer, (+)-1′-carba-dMGer, and (+)-6′-carba-dMGer with KAI2 and analyzed the mass spectrometry spectra under denaturing conditions; however, we were unable to detect any covalent adducts between the (+)-dMGer analogs and KAI2. The covalent bond formed between the catalytic triad of KAI2 and the (+)-dMGer hydrolyzate may be more unstable or transient than that formed between D14 and the SL hydrolyzate. Therefore, we synthesized the compound 2Br-PEO with a β-propiolactone ring instead of a butenolide ring, to investigate whether a covalent bond between the catalytic serine of KAI2 and the ligand hydrolyzate activated the receptor (*SI Appendix*, Fig. S13). The β-propiolactone ring was expected to be opened by nucleophilic attack of the catalytic serine to form a covalent bond with the residue. 2Br-PEO bound to the ligand-binding pocket of KAI2 with almost the same potency as (+)-dMGer and was hydrolyzed by KAI2 (*SI Appendix*, Fig. S13). Additionally, peptide matching from the MS/MS spectra identified a modified peptide of KAI2 with a molecular weight corresponding to C_11_H_12_O_2_Br covalently linked to Ser95 (*SI Appendix*, Fig. S13). However, in the *Arabidopsis* hypocotyl elongation assay, 2Br-PEO did not inhibit hypocotyl elongation and restored the GR24*^ent^*^-5DS^-induced inhibition (*SI Appendix*, Fig. S13); that is, 2Br-PEO acted as an antagonist rather than an agonist. These results indicated that 2Br-PEO did not induce a conformational change in KAI2, although it bound to the ligand-binding pocket and formed a covalent adduct with the catalytic serine. These results also suggested that a ligand interaction with the catalytic serine was not sufficient to activate the KAI2 protein. A similar result has been reported in studies with D14, which showed that β-lactone compounds are covalently bound to the catalytic serine and act as irreversible antagonists of D14 (54). Thus, the formation of covalent adducts with the catalytic histidine, but not with the serine, may be critical for the activation of both KAI2 and D14 (20, 43).

Most of the current understanding of the activation mechanism of KAI2 has come from analyses of receptor variants with mutated catalytic residues and the hydrolyzable ligand, GR24*^ent^*^-5DS^. The synthesis and characterization of (+)-carba-dMGer analogs in the present study have provided experimental evidence that ligand hydrolysis plays a key role in KAI2 signaling. In addition, our results suggested that ligand hydrolysis (i.e., ring opening) and covalent adduct formation with the catalytic serine are not sufficient for KAI2 activation. Although these results were not surprising, they indicate that the endogenous KAI2 ligand (KL) probably has a hydrolyzable butenolide ring. Furthermore, such carba-compounds with modified butenolide rings may also function as useful chemical tools in SL-signaling studies, where the function of the ligand hydrolysis remains controversial.

## Materials and Methods

### Materials.

The *Arabidopsis (A. thaliana) htl-3* allele (Col-0 ecotype) used here has been described previously (55). dMGer, 1′-carba-dMGer, 6′-carba-dMGer, and 2Br-PEO were synthesized, and the synthetic route is shown in *SI Appendix*, Figs. S2–S4. The detailed experimental data of these compounds are provided in *SI Appendix*, *Materials and Methods*.

### dYLG Assay.

The purified KAI2 (1 µg) was incubated with dYLG (0.5 µM) in the absence or presence of dMGer analogs for 10 min. Fluorescence intensity was measured using the Varioskan LUX plate reader (Thermo Fisher Scientific), with excitation and detection wavelengths of 480 and 520 nm, respectively. The IC_50_ values were calculated using the Enzyme Kinetics module of the SigmaPlot 14 program. Additional detailed methods are described in *SI Appendix*, *Materials and Methods*.

### DSF Assay.

DSF experiments were carried out using the StepOne real-time PCR system (Thermo Fisher Scientific). A Protein Thermal Shift™ Dye (Thermo Fisher Scientific) was used as the reporter dye. Reaction mixtures were prepared in PCR tubes, and each reaction was carried out on a 20 µL scale in KPi buffer containing 5 µg KAI2 protein, 2.5 µL Dye, and 1 µL DMSO solution of dMGer analogs. The denaturation curve was obtained using Protein Thermal Shift Software V1.4 (Thermo Fisher Scientific).

### Yeast Two-Hybrid Assay.

Y2H assays were performed as described previously (45) with some modification. Briefly, the coding sequences of KAI2 and SMAX1 were cloned into pGADT7 and pGBKT7, respectively. Resulting constructs were cotransformed with the yeast strainY2H Gold (Clontech) and the transformants were grown on SD−Trp/−Leu for 2 d at 30 °C. Interactions between the two proteins were examined on SD−Trp/−Leu/−His containing a 1,000 times dilution of dMGer analogs in acetone (0.1% acetone was used as a control). The plates were kept for 5 d at 30 °C.

### Hypocotyl Elongation Assay.

Hypocotyl elongation assays under red light were performed as described previously (6) with some modification. Surface-sterilized seeds were plated on solid 0.5 × MS media supplemented with test compounds or an equivalent volume of solvent [0.1% (v/v) DMSO] as indicated. Seeds were stratified in dark for 3 d at 2 °C and then moved to a CLE-305 growth chamber (TOMY Inc.) to grow at 22 °C under white light (25 µmol m^−2^ s^−1^) for 3 h, dark for 21 h. After that, the seeds were then transferred to a Plant Station LED Light BGA-V11S02RGB (Bio Medical Science Inc.) to grow at 22 °C under red light (640 nm, 20 µmol m^−2^ s^−1^) for 4 d. The hypocotyl lengths of the photographed seedlings were measured using ImageJ.

### Thermoinhibition Germination Assay of *Arabidopsis*.

The classic definition of radical emergence was used for seed germination assays. Approximately 30 to 50 seeds (Col-0) were sterilized by soaking in 70% aqueous ethanol (EtOH, v/v) for 30 min and reagent-grade EtOH for 1 min. The seeds were then socked in distilled water and incubated under continuous illumination at 32 °C for 3 d (thermoinhibition treatment). After 3 d, the seeds were transferred to normal conditions (continuous illumination at 22 °C) and the number of all seeds and germinated seeds was counted after another 2 to 5 d of incubation.

### Enzymatic Assay.

A reaction mixture containing purified KAI2 (3 µM) and (+)-/(−)-dMGer analogs or 2Br-PEO (10 µM final concentration) in PBS (pH 7.6) was incubated at 30 °C for 10 min. After adding 4-phenylphenol (0.3 nmol) as the internal standard, the residual (+)-dMGer analogs or 2Br-PEO were extracted with EtOAc (3 × 150 µL) and concentrated in *vacuo*. The dried sample was then dissolved in 50 µL of MeOH, after which a 10 µL aliquot was analyzed by HPLC. The HPLC conditions were as follows: column, Kinetex PS C_18_ (100 × 4.6 mm, 2.6 μm, Shimadzu GLC Ltd., Tokyo, Japan); solvent, 60% MeOH in H_2_O; flow rate, 1.5 mL min^−1^; and detection wavelength, 190−350 nm. Data are presented as the averages from three independent experiments. Additional detailed methods are described in *SI Appendix*, *Materials and Methods*.

### Crystallization, Data Collection, and Structure Determination.

Crystallization was performed using the sitting-drop vapor-diffusion method at 20 °C, and crystallization buffer comprised 0.1 M Tris-HCl (pH 7.0 to 9.0) with 1.25 to 2 M ammonium sulfate and 12% (v/v) glycerol as the reservoir buffer. Drops of protein solution (1.0 μL, 11 mg mL^−1^) were mixed with 1.0 μL of reservoir buffer. A crystal of KAI2 was dipped in the reservoir with 30% glycerol as a cryoprotectant and mounted on the goniometer. Diffraction of the 1.12 Å X-ray by the crystal was measured 360º with oscillation range 1º under 100 K. To solve the ligand complex structure, a crystal of KAI2 was soaked in the reservoir containing 100 μM (+)-6′-carba-dMGer for 2 h at 20 °C. The soaked crystal was dipped in the reservoir with 30% glycerol as a cryoprotectant. Diffraction of the 0.72 Å X-ray by the crystal was measured 360º with oscillation range 0.25º under 100 K. Additional detailed methods are described in *SI Appendix*, *Materials and Methods*.

### Mass Spectrometric Analysis of Covalent Modification with In-Gel Digestion.

Mass spectrometry analysis was performed using the previously described method (21) with some modifications. The complexes KAI2 with (+)-dMGer analogs were submitted to digestion by trypsin (Promega) and chymotrypsin (Promega) after reduction with DTT and alkylation with iodoacetamide. Additional detailed methods are described in *SI Appendix*, *Materials and Methods*.

## Supplementary Material

Appendix 01 (PDF)

## Data Availability

The atomic coordinates have been deposited in Protein Data Bank (PDB) under accession numbers 8ZVO (https://www.rcsb.org/structure/8ZVO) (56) for KAI2 apo structure; 8ZVN (https://www.rcsb.org/structure/8ZVN) (57) for KAI2-(+)-6’-carba-dMGer complex.
