# Supplementary material for: Structural requirements of KAI2 ligands for activation of signal transduction
Source: Proc Natl Acad Sci U S A. 2025 Feb 20;122(8):e2414779122. doi: 10.1073/pnas.2414779122 (PMC11874195; doi:10.1073/pnas.2414779122)
Supplement: Supplementary file 1 — Appendix 01 (PDF) [file pnas.2414779122.sapp.pdf]

Supplementary Information for

Structural requirements of KAI2 ligands for activation of signal transduction

Rito Kushihara[1], Akihiko Nakamura[2,3,4], Katsuki Takegami[1], Yoshiya Seto[5], Yusuke Kato[5], Hideo Dohra[3,6,7], Toshiyuki Ohnishi[2,3], Yasushi Todoroki[2,3], and Jun Takeuchi[2,3*]

Affiliations

[1]*Graduate School of Science and Technology, Shizuoka University, Shizuoka 422-8529, Japan*

[2]*Faculty of Agriculture, Shizuoka University, Shizuoka 422-8529, Japan*

[3]*Research Institute of Green Science and Technology, Shizuoka University, Shizuoka 422-8529, Japan*

[4]*Institute for Molecular Science, National Institutes of Natural Sciences, 5-1 Higashiyama Myodaijicho, Okazaki, Aichi 444-8787, Japan*

[5]*Laboratory of Plant Chemical Regulation, School of Agriculture, Meiji University, Kanagawa 214-8571, Japan*

[6]*Shizuoka Instrumental Analysis Center, Shizuoka University, Shizuoka 422-8529, Japan*

[7]*Graduate School of Integrated Science and Technology, Shizuoka University, Shizuoka 422-8529, Japan*

Corresponding author: Jun Takeuchi
Email: takeuchi.jun@shizuoka.ac.jp

This PDF file includes:

Figures S1 to S23
Table S1
Supplementary text
SI References

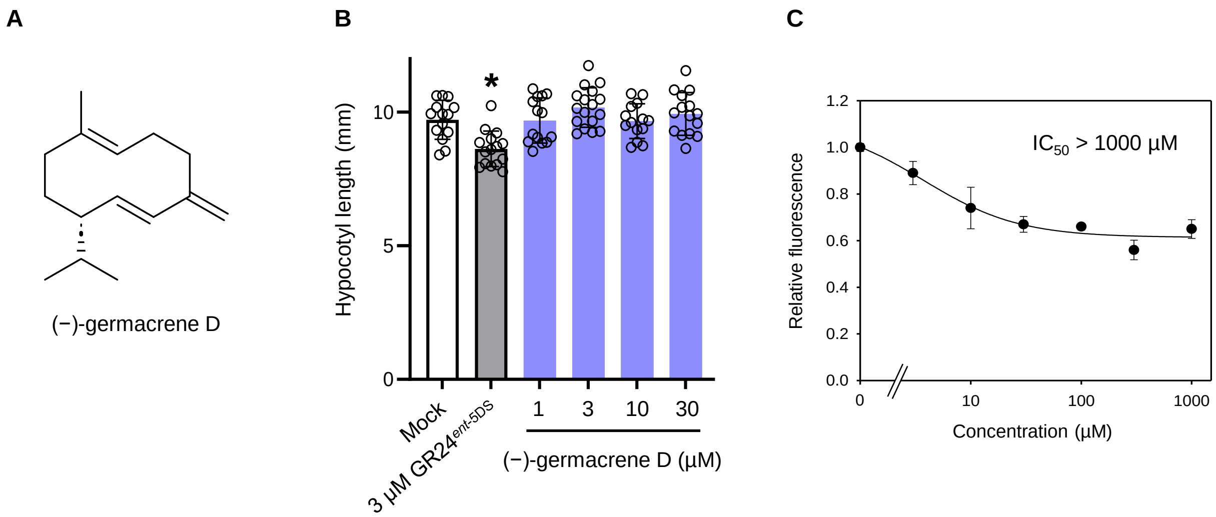

Supplementary Figure S1. Effects of (−)-germacrene D on *Arabidopsis* KAI2 (A) Structure of (−)-germacrene D. (B) *Arabidopsis* (Col-0) hypocotyl elongation response to (−)-germacrene D. Data are the means ±SD (n = 13–15). Asterisks indicate a significant difference compared with mock-treated seedlings: *, $P < 0.05$ (Dunnett's test). Small circles indicate each data point. Similar results were obtained in two independent experiments. (C) Inhibitory effects of (−)-germacrene D on KAI2 hydrolytic activity. Relative fluorescence of the dYLG probe in the presence of purified KAI2 and increasing concentrations of (−)-germacrene D (n = 3; error bars represent the SDs). The listed IC_{50} values are the averages from three experiments.

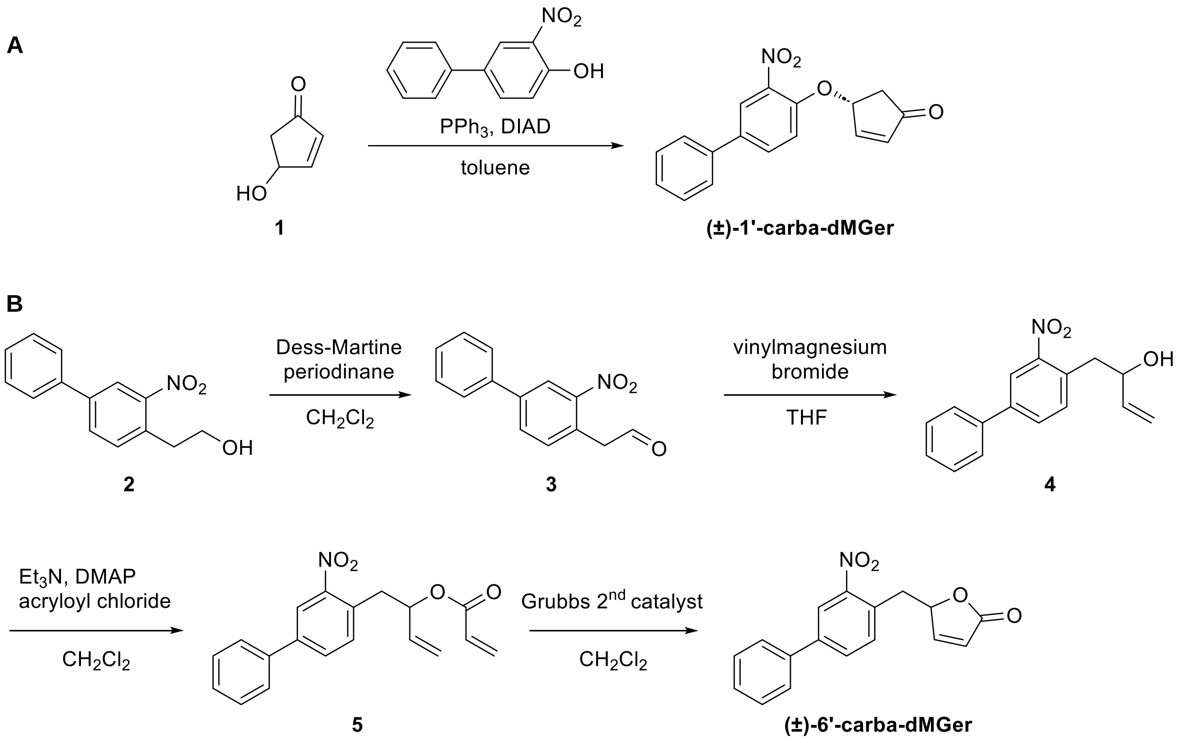

Supplementary Figure S2. Synthesis of (±)-1'-carba-dMGer (A) and (±)-6'-carba-dMGer (B).

A

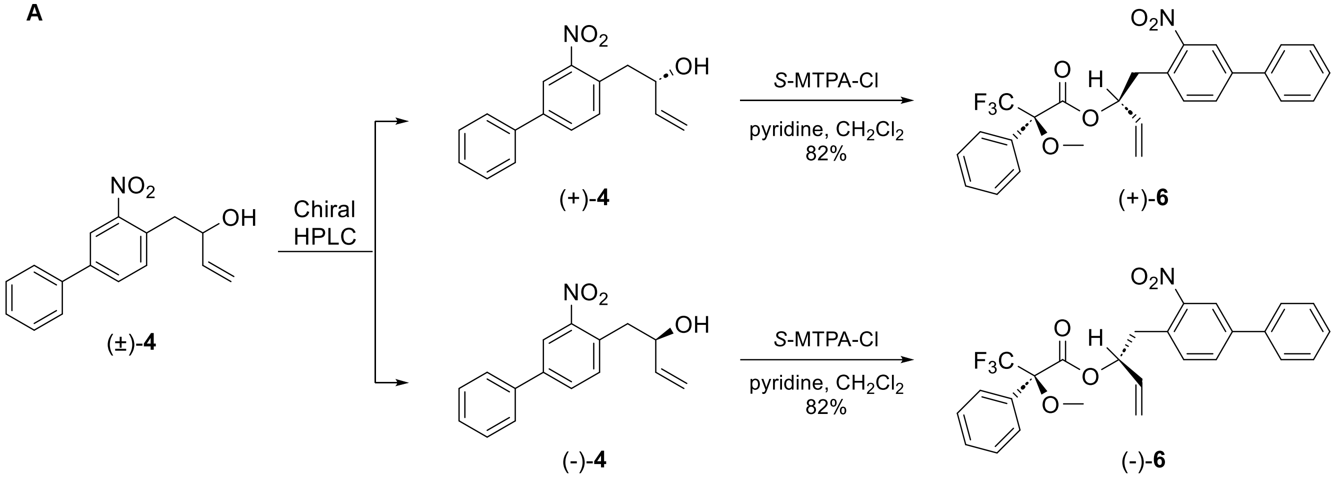

B

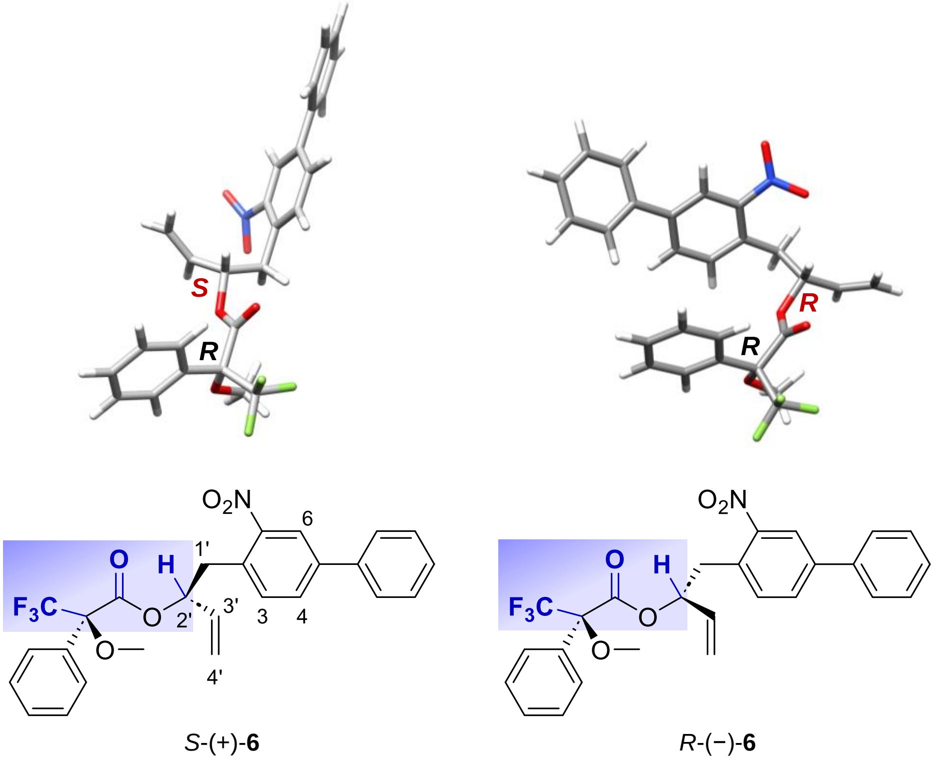

H	δ (ppm) *R*-MTPA ester of (+)-**6**	(-)-**6**
2'	5.81	5.84
1'	3.23	**3.19**
1'	3.48	**3.43**
3'	**5.88**	5.95
4'	**5.30**	5.35
4'	**5.39**	5.47
3	7.36	**7.16**
4	7.68	**7.51**
6	8.26	**8.19**

$\Delta\delta = \delta_S - \delta_R$ (ppm)

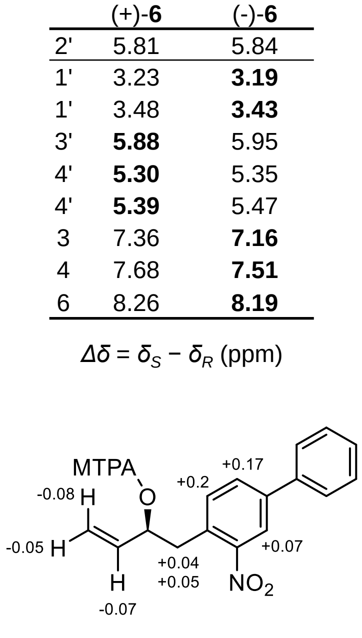

Supplementary Figure S3. Determination of the absolute configuration of optically pure compound **4** by an advanced Mosher's method. (A) Synthesis of MTPA esters of (+)-/(−)-**4**. (B) Molecular models of the MTPA esters were optimized with B3LYP/6-31G(d) from the initial geometries with the ideal MTPA plane. Stick models: carbons, gray; hydrogens, white; oxygens, red; nitrogen, blue; and fluorines, light green. The structural formulas correspond to views from the right side of the models. The MTPA plane, defined by the trifluoromethyl group, the carbonyl group, and the carbinol proton, are colored in purple in the structural formula.

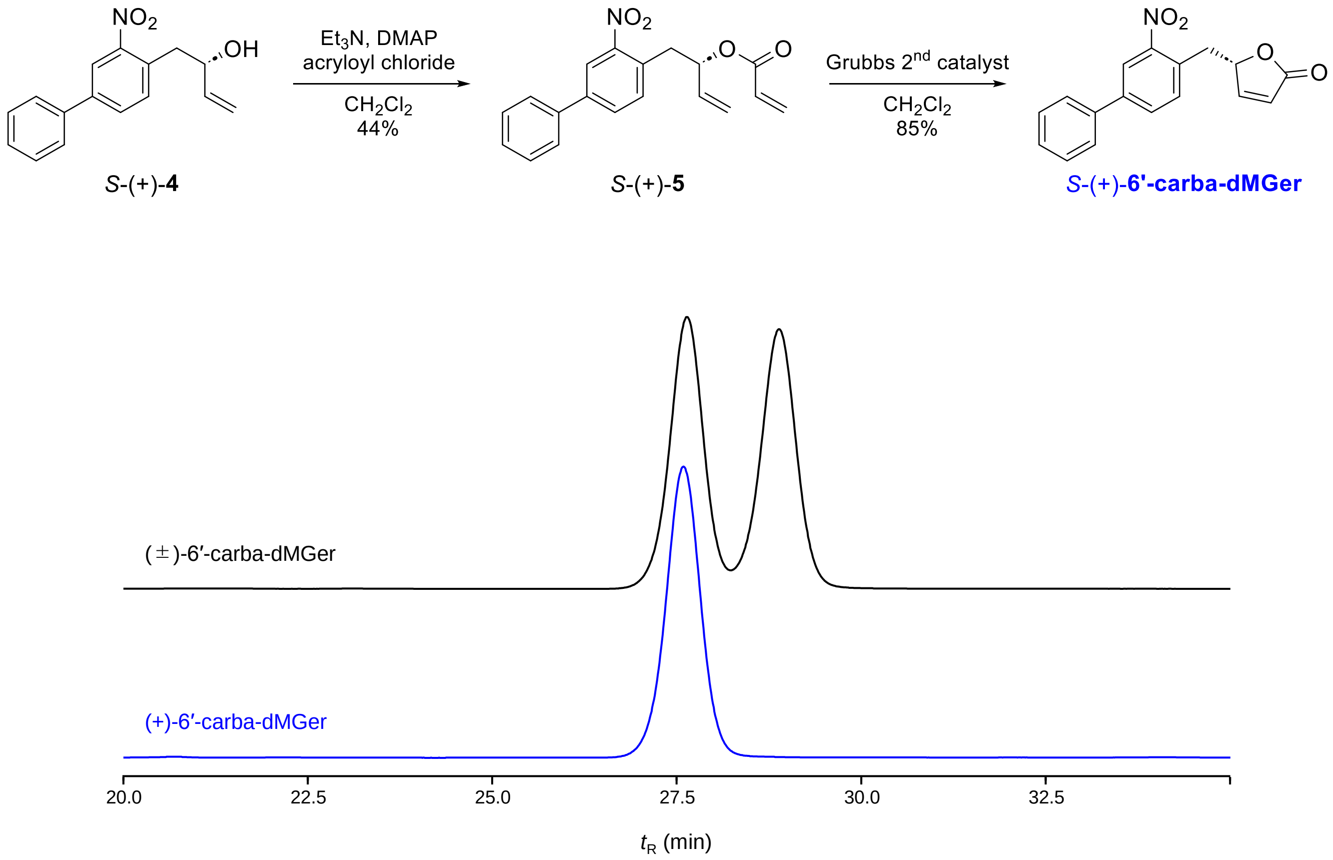

Supplementary Figure S4. Synthesis of *S*-(+)-6′-carba-dMGer and its chiral HPLC chromatogram. HPLC conditions were as follows: column, CHIRAL ART Cellulose-SC (250 × 4.6 mm, 5 µm, YMC); solvent, 20% EtOAc in hexane; flow rate, 0.5 mL min^{-1}; and detection wavelength, 254 nm. The retention times of (+)-6′-carba-dMGer and its (−)-isomer are 27.6 and 28.9 min, respectively.

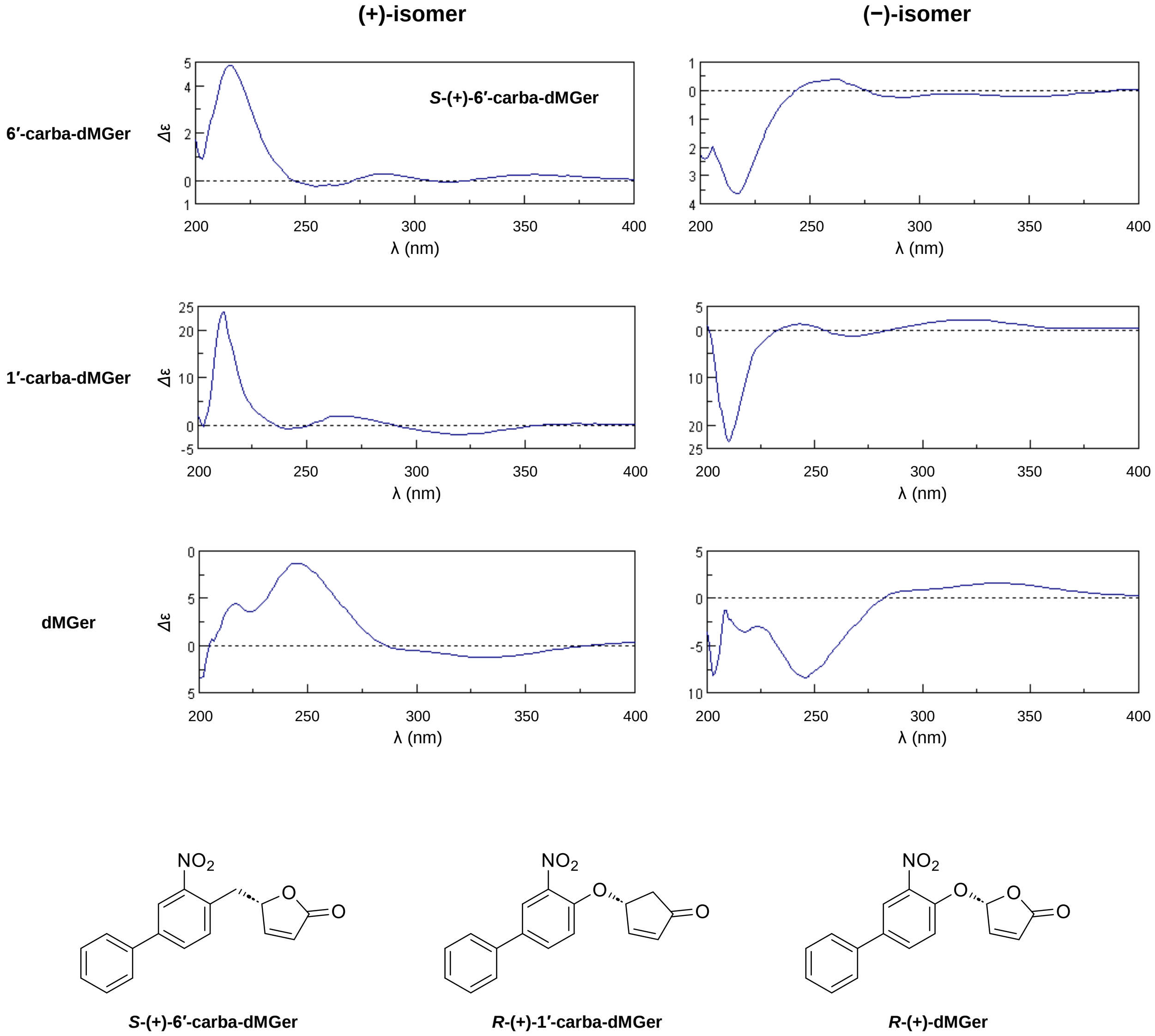

Supplementary FigureS5. Experimental CD spectra of enantiomers of 6′-carba-dMGer, 1′-carba-dMGer, and dMGer and structures of *S*-(+)-6′-carba-dMGer, *R*-(+)-1′-carba-dMGer, and *R*-(+)-dMGer.

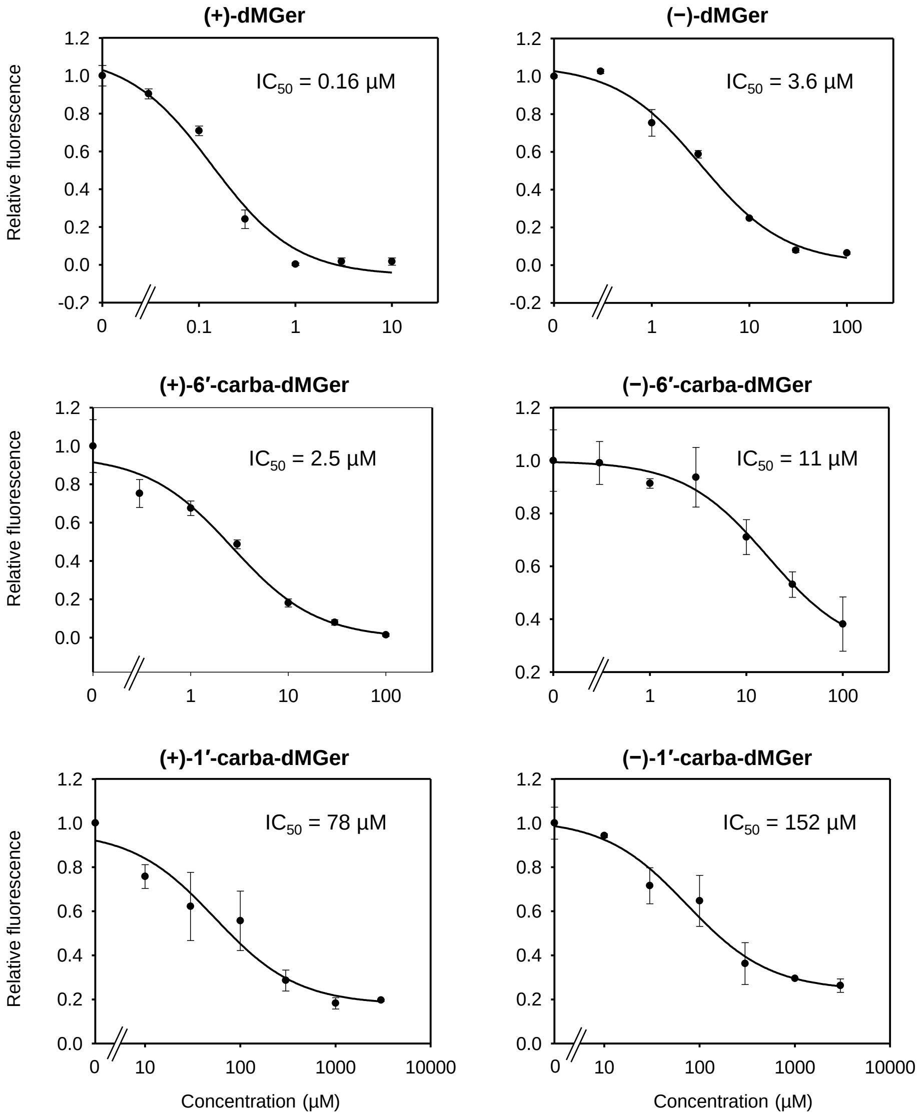

Supplementary Figure S6. Inhibitory effects of dMGer analogs on KAI2 hydrolytic activity. Relative fluorescence of the dYLG probe in the presence of purified KAI2 and increasing concentrations of (+)-dMGer, (−)-dMGer, (+)-6′-carba-dMGer, (−)-6′-carba-dMGer, (+)-1′-carba-dMGer or (−)-1′-carba-dMGer (*n* = 3; error bars represent the SDs). The listed IC_{50} values are the averages from three experiments.

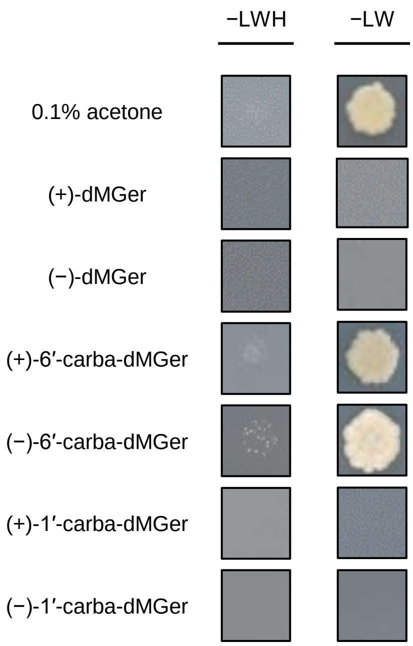

Supplementary Figure S7. Analysis of the KAI2-SMAX1 interaction in the presence of (+)-/(−)-dMGer, (+)-/(−)-6′-carba-dMGer, or (+)-/(−)-1′-carba-dMGer using the yeast-two hybrid method. Yeast transformants were spotted onto the selective medium [SD−Leu/−Trp/−His (−LWH)] in the absence or presence of 10 µM dMGer analogs.

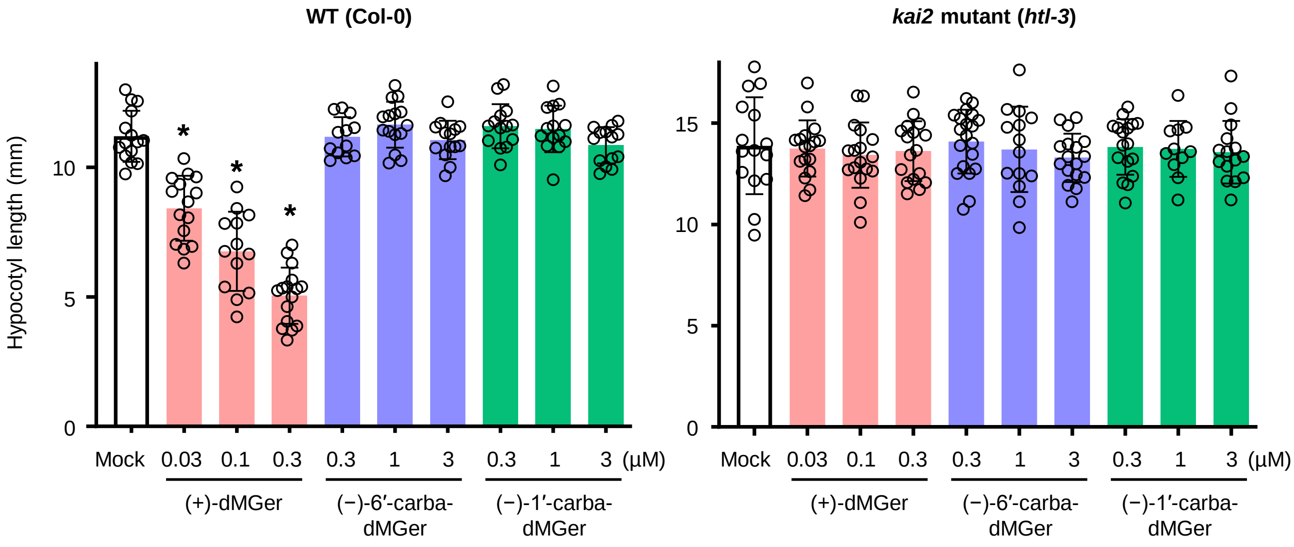

Supplementary Figure S8. *Arabidopsis* (Col-0) hypocotyl elongation response to (−)-6′-carba-dMGer and (−)-1′-carba-dMGer in WT and *kai2* mutant (*htl-3*). Data are the means ± SD (n = 13–16). Asterisks indicate a significant difference compared with mock-treated seedlings: * $P < 0.05$ (Dunnett's test). Small circles indicate each data point. Similar results were obtained in two independent experiments.

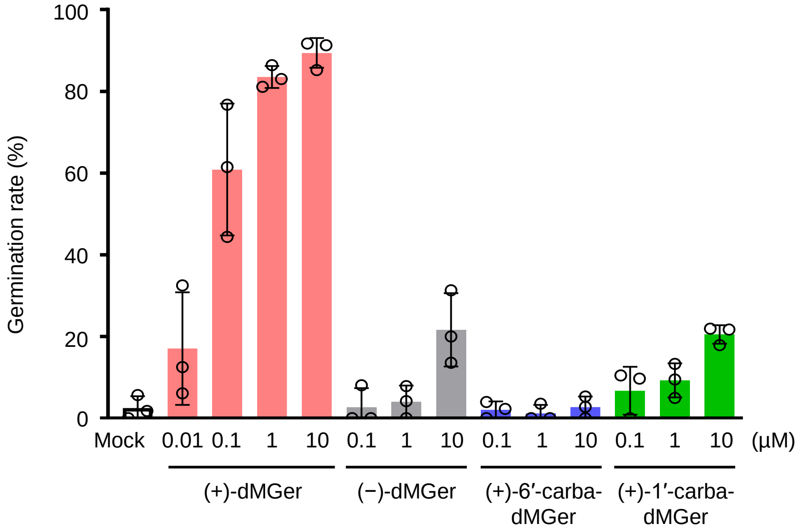

Supplementary Figure S9. Seed germination rate in the presence of the indicated concentrations of (+)-dMGer, (−)-dMGer, (+)-6′-carba-dMGer, and (+)-1′-carba-dMGer. *Arabidopsis* (Col-0) seeds (30–50 seeds/well) were imbibed with sterilized distilled water containing each chemical, or DMSO as a control, and incubated at 32 °C with continuous light for 2 days (thermoinhibition treatment). After 2 days of thermoinhibition treatment, the seeds were transferred to the ordinary condition (22 °C, continuous light), and the numbers of all seeds and the germinated seeds (radicle emergence) were counted after an additional 4 days of incubation. Data are the means ± SD (n = 3). Similar results were obtained in two independent experiments.

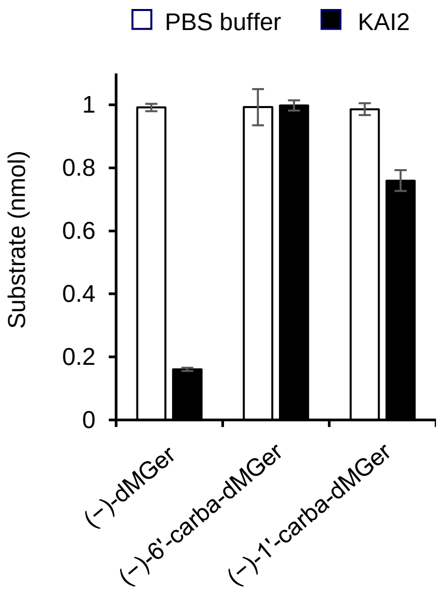

Supplementary Figure S10. Enzymatic degradation of (−)-dMGer, (−)-6′-carba-dMGer, and (−)-1′-carba-dMGer by KAI2. Each test compound (10 µM) was incubated with or without KAI2 (3 µM) (n = 3; error bars represent the SDs.).

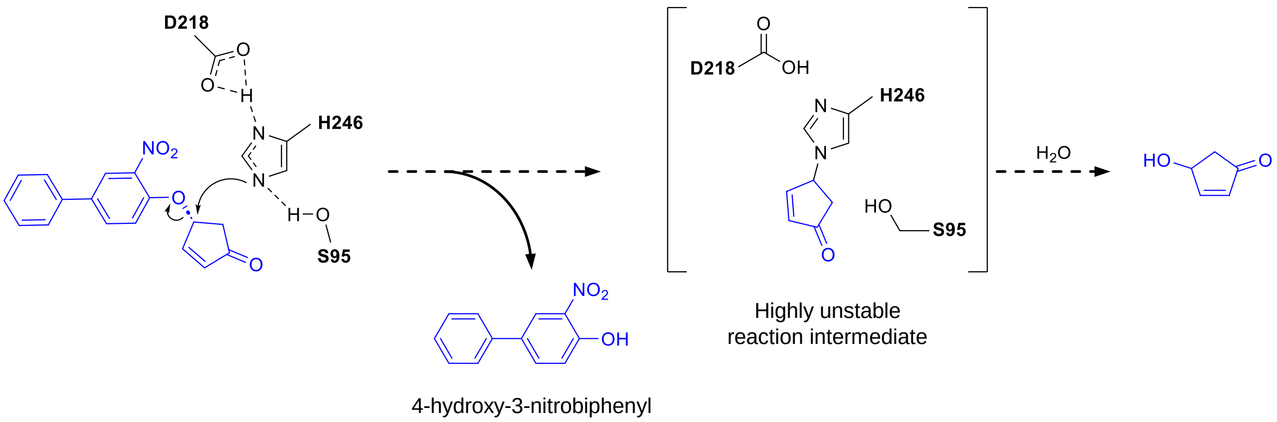

Supplementary Figure S11. Predicted schematic diagram of the hydrolysis pathway of (+)-1′-carba-dMGer by KAI2. The process begins with the nucleophilic attack of the NE2 atom of His246 on the 2′C atom of (+)-1′-carba-dMGer, which in turn releases a 4-hydroxy-3-nitrobiphenyl. Dotted arrows indicate a hypothetical step.

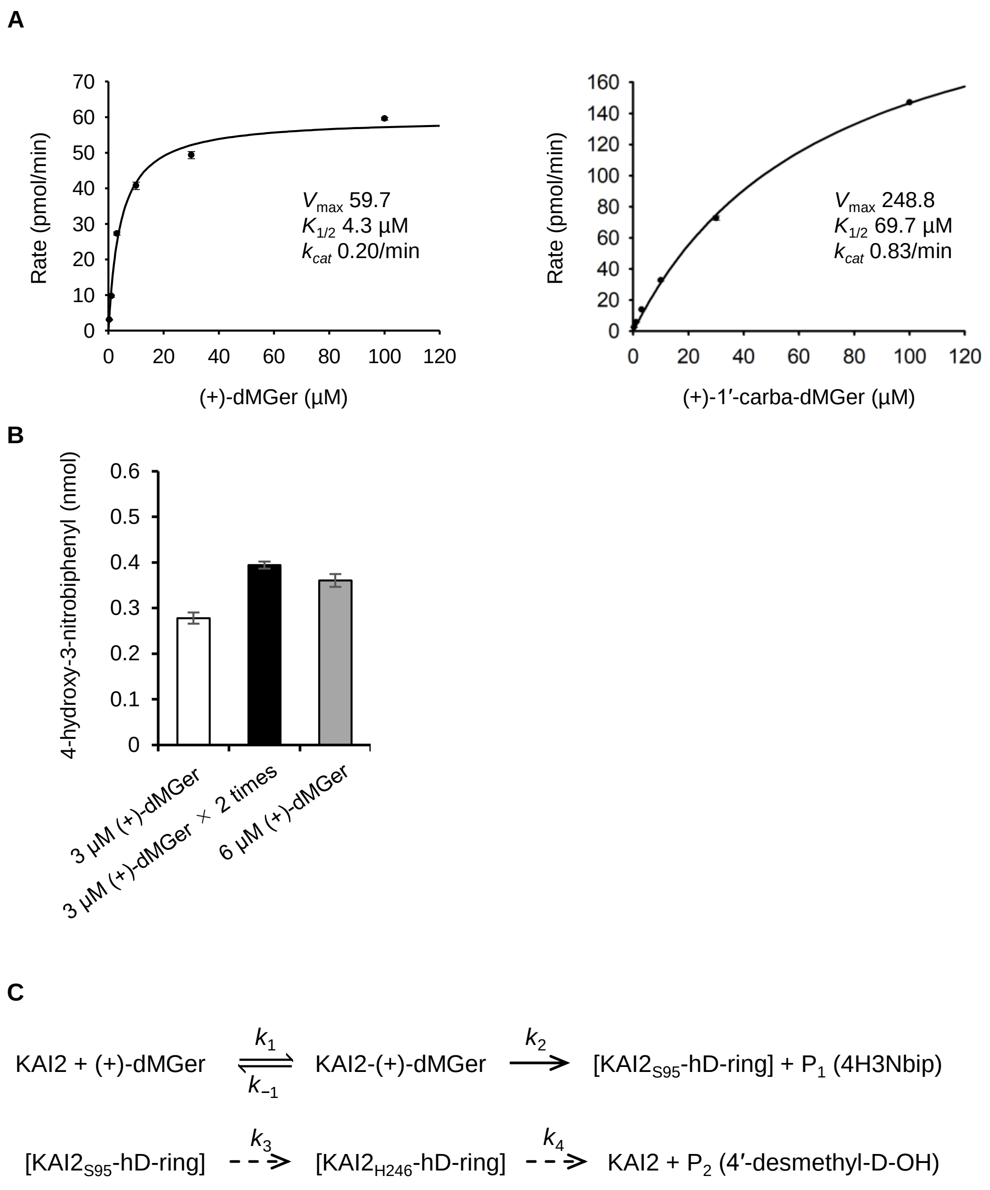

Supplementary Figure S12. Enzymatic kinetics indicated that the dissociation of the hydrolyzed furanone ring of (+)-dMGer from the KAI2 ligand binding pocket was very slow. (A) Hyperbolic plot of the KAI2 pre-steady-state kinetics reaction velocity with (+)-dMGer (left) or (+)-1′-carba-dMGer (right). Enzymatic activity was defined as the amount of 4-hydroxy-3-nitrobiphenyl produced over 10 min in the presence of 3 µM KAI2 protein and 0.3, 1, 3, 10, 30, or 100 µM (+)-dMGer or (+)-1′-carba-dMGer. (B) Inhibition of the hydrolysis of (+)-dMGer by the hydrolyzed furanone ring of (+)-dMGer. After completion of the hydrolysis of (+)-dMGer (3 µM) by KAI2 protein (3 µM), (+)-dMGer (3 µM) was successively added to the reaction mixture and the hydrolyzed product (4-hydroxy-3-nitrobiphenyl) was quantified. Each error bar represents the SDs of three replicates. (C) Proposed kinetic mechanism for KAI2 to recognize (+)-dMGer. Double arrows indicate equilibrium and single arrows an irreversible reaction. Dotted arrows indicate a hypothetical step.

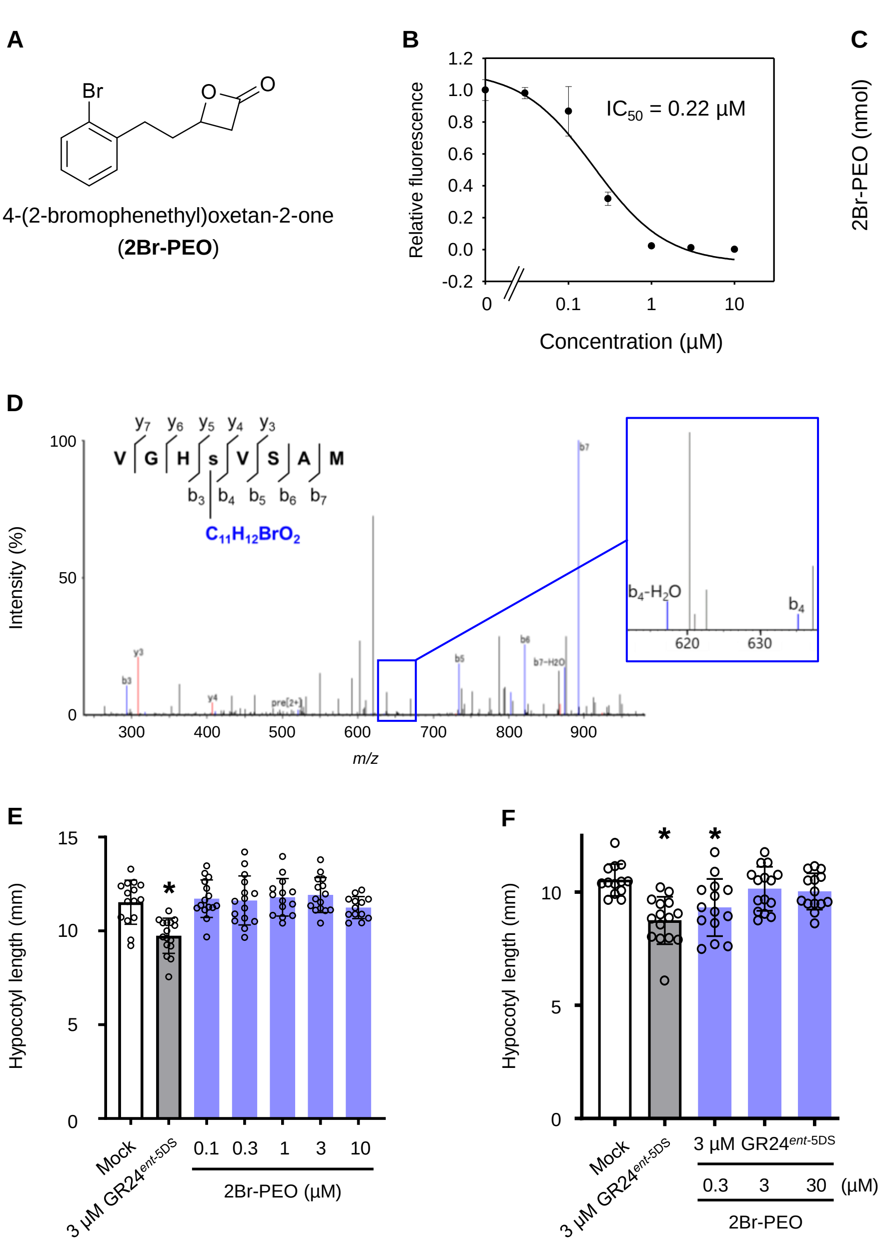

Supplementary Figure S13. A β-propiolactone compound covalently bound to the catalytic Ser95 of KAI2 but did not induce KAI2 signal transduction. (A) Structure of the β-propiolactone compound, 2Br-PEO. (B) Inhibitory effect of 2Br-PEO on the hydrolytic activity of KAI2. Relative fluorescence of the dYLG probe in the presence of purified KAI2 and increasing concentrations of 2Br-PEO. The listed IC_{50} values are the averages from three experiments. (C) Enzymatic degradation of 2Br-PEO by KAI2. 2Br-PEO (10 µM) was incubated with or without KAI2 (3 µM) (n = 3; error bars represent the SDs). (D) Identification of the 2Br-PEO-induced $C_{11}H_{12}BrO_2$ modification of S95 of KAI2. MS/MS spectra of a double charged peptide (92-VGHsVSAM-99) of KAI2 at mass-to-charge (*m/z*) 521.2265 corresponding to the mass of the $C_{11}H_{12}BrO_2$ modification of S95. (E) *Arabidopsis* (Col-0) hypocotyl elongation response to 2Br-PEO. (F) *Arabidopsis* (Col-0) hypocotyl elongation after treatment with 3 µM GR24$^{ent\text{-}5DS}$ and 2Br-PEO. (E, F) Data are the means ± SD (n = 13–16). Asterisks indicate a significant difference compared with mock-treated seedlings: * $P < 0.05$ (Dunnett's test). Similar results were obtained in two independent experiments.

Table S1. Data collection, phasing and refinement statistics.

	AtKAI2 (apo form)	AtKAI2-(+)-6'-carba-dMGer
PDB ID	8ZVO	8ZVN
Wavelength	1.12	0.72
Resolution range	43.86 - 1.49 (1.543 - 1.49)	36.46 - 2.08 (2.154 - 2.08)
Space group	P 1 21 1	C 1 2 1
Unit cell	50.7 55.79 52.87 90 115.94 90	88.32 55.97 53.19 90 98.91 90
Total reflections	313953 (29036)	106285 (10283)
Unique reflections	42702 (4105)	15363 (1522)
Multiplicity	7.4 (7.1)	6.9 (6.8)
Completeness (%)	98.31 (94.76)	98.82 (98.32)
Mean I/sigma(I)	26.43 (5.51)	13.90 (3.30)
Wilson B-factor	12.72	25.2
R-merge	0.04876 (0.3835)	0.1012 (0.522)
R-meas	0.0525 (0.4136)	0.1094 (0.5656)
R-pim	0.01931 (0.1535)	0.04124 (0.215)
CC1/2	0.999 (0.953)	0.998 (0.935)
CC*	1 (0.988)	1 (0.983)
Reflections used in refinement	42702 (4104)	15363 (1520)
Reflections used for R-free	2001 (195)	1536 (153)
R-work	0.1465 (0.2063)	0.1602 (0.2676)
R-free	0.1724 (0.2309)	0.2290 (0.3292)
CC(work)	0.973 (0.928)	0.972 (0.932)
CC(free)	0.967 (0.896)	0.935 (0.811)
Number of non-hydrogen atoms	2632	2402
macromolecules	2207	2172
ligands	59	64
solvent	366	179
Protein residues	268	268
RMS(bonds)	0.007	0.007
RMS(angles)	0.91	0.83
Ramachandran favored (%)	98.5	97.74
Ramachandran allowed (%)	1.5	2.26
Ramachandran outliers (%)	0	0
Rotamer outliers (%)	0	2.46
Clashscore	5.31	10.46
Average B-factor	17.93	33.76
macromolecules	15.43	33.25
ligands	32.64	38.56
solvent	30.63	38.53

Experimental

General procedures

^{1}H NMR spectra were recorded with tetramethylsilane as the internal standard using JNM-ECZ R (500 MHz) NMR spectrometers (JEOL Ltd., Tokyo, Japan). For peak assignments, refer to the numbering of the structures of dMGer (Fig. 1). High resolution mass spectra were obtained with a JEOL JMS-T100LC AccuTOF mass spectrometer (ESI-TOF, positive mode; JEOL Ltd.). Optical rotations were recorded with a Jasco DIP-1000 digital polarimeter. Circular dichroism spectra were recorded with a Jasco J-820 spectrophotometer. Column chromatography was performed using silica gel (Wakosil 60, Wako P22ure Chemical Industries, Ltd.). (−)-Germacrene D was purchased from Cayman Chemical.

Synthesis of (±)-dMGer analogs

4-((3-nitro-[1,1'-biphenyl]-4-yl)oxy)cyclopent-2-en-1-one, (±)-1′-carba-dMGer

4-hydroxy-3-nitrobiphenyl (97 mg, 0.45 mmol) and triphenylphosphine (134 mg, 0.51 mmol) under an atmosphere of argon were added to a solution of **1** (40 mg, 0.41 mmol) in toluene (2 mL). After stirring the mixture for 10 min at −10 °C, diisopropyl azodicarboxylate (210 μL, 0.49 mmol) was added dropwise to the mixture. The reaction mixture was stirred for 1 h at the same temperature. After quenching with sat $NaHCO_3$ solution (5 mL), it was then extracted with EtOAc (10 ml × 3), washed with brine, dried over Na_2SO_4, and concentrated *in vacuo*. The residual oil was purified by silica gel chromatography with 5% EtOAc in toluene to obtain (±)-**1′-carba-dMGer** (38 mg, 32%) as a yellow oil. ^{1}H NMR (500 MHz, $CDCl_3$): δ_H 2.60 (1H, dd, *J*=18.2 and 2.3 Hz, H-1′), 2.98 (1H, dd, *J*=18.2 and 6.2 Hz, H-1′), 5.60 (1H, m, H-2′), 6.45 (1H, dd, *J*=5.7 and 1.1 Hz, H-4′), 7.21 (1H, d, *J*=8.6 Hz, H-3), 7.40 (1H, m, H-10), 7.48 (2H, m, H-9 and 11), 7.56 (2H, m, H-8 and 12), 7.75 (1H, dd, *J*=5.7 and 2.3 Hz, H-3′), 7.79 (1H, dd, *J*=8.6 and 2.3 Hz, H-4), 8.09 (1H, d, *J*=2.3 Hz, H-6); ^{13}C NMR (126 Hz, $CDCl_3$): δc 41.6, 77.5, 116.2, 124.3, 126.8, 126.8, 128.2, 129.1, 129.1, 132.3, 135.4, 137.3, 138.0, 141.0, 149.8, 158.1, 203.8;HRMS (*m/z*): $[M+Na]^+$ calcd for $C_{17}H_{13}NO_4Na$, 318.0737; found, 318.0518

A CHIRAL ART Cellulose-SZ HPLC column (250 × 10.0 mm i.d., YMC; solvent, 45% isopropanol in hexane; flow rate, 2.5 mL min^{-1}; detection, 254 nm) was injected with (±)-**1′-carba-dMGer**. The material at t_R 23.4 and 25.1 min were collected to give (+)-**1′-carba-dMGer** and the (−)-enantiomer with optical purity of 99.8% and 97.3%, respectively. (+)-**1′-carba-dMGer**: $[\alpha]_D^{20}$ +33.8 (MeOH; *c* 0.20). (−)-**1′-carba-dMGer**: $[\alpha]_D^{20}$ −30.8 (MeOH; *c* 0.20).

2-(3-nitro-[1,1'-biphenyl]-4-yl)ethan-1-ol (2)

Paraformaldehyde (121 mg, 4.03 mmol), 4-methyl-3-nitro-1,1'-biphenyl(1) (1.29 g, 6.03 mmol) and Triton-B (73 μL, 40% MeOH) were dissolved in DMSO (7.3 mL). The resulting mixture was heated 2 h at 90 °C. After cooling, the reaction mixture was diluted with a saturated solution of NH_4Cl, it was extracted with EtOAc (15 mL × 3). The organic layer was washed with brine, dried over Na_2SO_4, and

concentrated *in vacuo*. The residual oil was purified by flash chromatography (10–40% EtOAc in hexane) to obtain **2** (552 mg, 56%) as a brown oil. ^{1}H NMR (500 MHz, $CDCl_3$): δ_H 3.21 (2H, t, *J*=6.3 Hz), 3.99 (2H, t, *J*=6.3 Hz), 7.42 (1H, m), 7.46–7.50 (3H, m), 7.58–7.60 (2H, m), 7.77 (1H, dd, *J*=8.0 and 1.7 Hz), 8.16 (1H, d, *J*=1.7 Hz). The data were consistent with the previous data(2).

2-(3-nitro-[1,1'-biphenyl]-4-yl)acetaldehyde

To a solution of alcohol **2** (652 mg, 2.68 mmol) in CH_2Cl_2 (5 mL) was added Dess-Martin periodinane (1.43 g, 3.37 mmol). After stirring the mixture for 3 h at room temperature, the reaction mixture was filtered through a pad of Celite and washed with sat. aqueous $NaHCO_3$ solution. The organic layer was dried over Na_2SO_4 and concentrated *in vacuo*. The crude was fractionated by flash chromatography (14–35% EtOAc in hexane) to obtain **3** (373 mg, 58%) as an orange oil. ^{1}H NMR (500 MHz, $CDCl_3$): δ_H 4.16 (2H, s, -*CH$_2$*CHO), 7.39 (1H, d, J=8.0 Hz, H-3), 7.42 (1H, m, H-10), 7.50 (2H, m, H-9 and 11), 7.62 (2H, m, H-8 and 12), 7.84 (1H, dd, *J*=8.0 and 1.7 Hz, H-4), 8.36 (1H, d, *J*=1.7 Hz, H-6), 9.89 (1H, s, -C*H*O); ^{13}C NMR (126 MHz, $CDCl_3$): δc 48.2, 123.7, 126.9, 127.0, 127.0, 128.6, 129.1, 129.2, 132.12, 133.9, 138.1, 142.3, 149.2, 196.8; HRMS (*m/z*): $[M+Na]^+$ calcd for $C_{14}H_{11}NO_3Na$ 264.0631; found 264.0271.

1-(3-nitro-[1,1'-biphenyl]-4-yl)but-3-en-2-ol (4)

Aldehyde **6** (373 mg, 1.55 mmol) in anhydrous THF (3 mL) was cooled to 0 °C. Vinylmagnesium bromide (1 M solution in THF, 2.4 mL, 2.4 mmol) was then added slowly. The reaction mixture was stirred for further 3 h at the same temperature. After quenching with sat. solution of NH_4Cl (5 mL), it was extracted with EtOAc (15 mL × 3). The organic layer was washed with brine, dried over $Na2_{S}O_4$, and concentrated *in vacuo*. The residual oil was purified by silica gel chromatography with 5% EtOAc in toluene to obtain **4** (70 mg, 17%) as a yellow oil. ^{1}H NMR (500 MHz, $CDCl_3$): δ_H 3.09 (1H, dd, *J*=13.8 and 5.8 Hz, -*CH$_2$*CHOH-), 3.27 (1H, dd, *J*=13.8 and 4.0 Hz, -*CH$_2$*CHOH-), 4.49 (1H, m, -C*H*OH), 5.18 (1H, ddd, *J*=10.3, 1.1 and 1.1 Hz, -CH=*CH$_2$*), 5.30 (1H, ddd, *J*=17.2, 1.1 and 1.1 Hz, -CH=*CH$_2$*), 6.00 (1H, ddd, *J*=17.2, 10.3 and 5.7 Hz, -*CH*=CH_2), 7.42 (1H, m, H-10), 7.47–7.50 (3H, m, H-3, 9 and 11), 7.60 (2H, m, H-8 and 12), 7.77 (1H, dd, *J*=8.0 and 1.7 Hz, H-4), 8.15 (1H, d, *J*=1.7 Hz, H-6); ^{13}C NMR (126 MHz, $CDCl_3$): δc 40.1, 73.0, 115.4, 123.1, 127.0, 127.0, 128.4, 129.1, 129.1, 131.1, 131.7, 133.9, 138.4, 140.0, 141.0, 150.2; HRMS (*m/z*): $[M+Na]^+$ calcd for $C_{16}H_{15}NO_3Na$ 292.0944; found 292.0736.

A CHIRAL ART Cellulose-SZ HPLC column (250 × 10.0 mm i.d., YMC; solvent, 5% EtOAc in hexane; flow rate, 2.5 mL min^{-1}; detection, 254 nm) was injected with (±)-**4**. The material at t_R 18.2 and 21.2 min were collected to give (+)-**4** and the (−)-enantiomer with optical purity of 99.2% and 97.5%, respectively. (+)-**7**: $[\alpha]_D^{22}$ +42.5 (MeOH; *c* 0.20). (−)-**4**: $[\alpha]_D^{22}$ −34.0 (MeOH; *c* 0.20).

1-(3-nitro-[1,1'-biphenyl]-4-yl)but-3-en-2-yl acrylate (5)

Et_3N (44 µL, 0.26 mmol), dimethylaminopyridine (DMAP; 2.3 mg, 0.02 mmol) and acryloyl chloride (21 µL, 0.26 mmol) were added to a cooled (0 °C) solution of alcohol **4** (70 mg, 0.26 mmol) in CH_2Cl_2 (2.8 mL). The reaction mixture was stirred for further 1 h at 0 °C. After quenching with water (10 mL), it was extracted with EtOAc (10 mL × 3). The organic layer was washed with brine, dried over Na_2SO_4, and concentrated *in vacuo*. The residual oil was purified by flash chromatography (6–27% EtOAc in hexane) to obtain **5** (20 mg, 24%) as a pale-yellow oil. ^{1}H NMR (500 MHz, $CDCl_3$): δ_H 3.29 (1H, dd, *J*=13.8 and 8.0 Hz, H-1′), 3.44 (1H, dd, *J*=13.8 and 4.6 Hz, H-1′), 5.23 (1H, d, *J*=10.3 Hz, H-4′), 5.31 (1H, d, *J*=17.2 Hz, H-4′), 5.66 (1H, m, H-2′), 5.81 (1H, dd, *J*=10.3 and 1.7 Hz, H-7′), 5.91 (1H, ddd, *J*=17.2, 10.3 and 5.7 Hz, H-3′), 6.08 (1H, dd, *J*=17.2 and 10.3 Hz, H-6′), 6.36 (1H, dd, *J*=17.2 and 1.7 Hz, H-7′), 7.39–7.43 (2H, m, H-3 and 10), 7.46–7.49 (2H, m, H-9 and 11), 7.60 (2H, m, H-8 and 12), 7.73 (1H, dd, *J*=8.0 and 1.7 Hz, H-4), 8.15 (1H, d, *J*=1.7 Hz, H-6); ^{13}C NMR (126 MHz, $CDCl_3$): δc 37.3, 74.5, 117.5, 123.2, 126.9, 126.9, 128.2, 128.4, 129.1, 129.1, 130.5, 131.0, 131.1, 133.5, 135.3, 138.2, 141.2, 150.2, 165.1; HRMS (*m/z*): $[M+Na]^+$ calcd for $C_{19}H_{17}NO_4Na$ 346.1050; found 346.0874.

(*S*)-1-(3-nitro-[1,1'-biphenyl]-4-yl)but-3-en-2-yl acrylate, (+)-5

Compound (+)-**4** (30.5 mg, 113 µmol) was converted to *S*-(+)-**5** (16.1 mg, 50 µmol, 44%) by the same manner. $[\alpha]_D^{22}$ +59.7 (MeOH; *c* 0.20).

5-((3-nitro-[1,1'-biphenyl]-4-yl)methyl)furan-2(5H)-one, (±)-6′-carba-dMGer

A CH_2Cl_2 solution (1 ml) of branched allylic ester **5** (20 mg, 0.062 mmol) was added to a solution of a second-generation Grubbs catalyst (3.2 mg, 6 mol%) in CH_2Cl_2 (4 ml). The reaction mixture was stirred under reflux for 3 h. After cooling to room temperature, it was quenched with water (5 mL) and extracted with CH_2Cl_2 (10 mL × 3). The organic layer was washed with brine, dried over Na_2SO_4, and concentrated *in vacuo*. The residual oil was purified by flash chromatography (31–52% EtOAc in hexane) to obtain **(±)-6′-carba-dMGer** (10.6 mg, 58%) as a brown oil. ^{1}H NMR (500 MHz, $CDCl_3$): δ_H 3.21 (1H, dd, *J*=14.4 and 8.0 Hz, H-6′), 3.55 (1H, dd, *J*=14.4 and 4.6 Hz, H-6′), 5.47 (1H, m, H-2′), 6.14 (1H, dd, *J*=5.7 and 1.7 Hz, H-4′), 7.43 (1H, m, H-10), 7.49 (2H, m, H-9 and 11), 7.52 (1H, d, *J*=8.0 Hz, H-3), 7.58–7.61 (3H, m, H-8, 12 and 3′), 7.81 (1H, dd, *J*=8.0 and 2.3 Hz, H-4), 8.24 (1H, d, *J*=2.3 Hz, H-6); ^{13}C NMR (126 MHz, $CDCl_3$): δc 36.6, 82.4, 122.3, 123.4, 127.0, 127.0, 128.6, 129.1, 129.2, 129.2, 131.7, 134.2, 138.0, 142.0, 149.4, 155.3, 172.4; HRMS (*m/z*): $[M+Na]^+$ calcd for $C_{17}H_{13}NO_4Na$ 318.0737; found 318.0506.

A CHIRAL ART Cellulose-SC HPLC column (250 × 10.0 mm i.d., YMC; solvent, 20% EtOAc in hexane; flow rate, 2.5 mL min^{-1}; detection, 254 nm) was injected with (±)-**6′-carba-dMGer**. The material at t_R 28.7 and 31.1 min were collected to give (+)-**6′-carba-dMGer** and the (−)-enantiomer with optical purity of 99.8% and 99.1%, respectively. (+)-**6′-carba-dMGer**: $[\alpha]_D^{23}$ +69.2 (MeOH; *c* 0.10). (−)-**6′-carba-dMGer**: $[\alpha]_D^{23}$ −64.6 (MeOH; *c* 0.10).

(*S*)-5-((3-nitro-[1,1'-biphenyl]-4-yl)methyl)furan-2(5H)-one, (+)-6′-carba-dMGer

Compound (+)-**8** (13.6 mg, 42 µmol) was converted to *S*-(+)-**6′-carba-dMGer** (10.6 mg, 36 µmol, 85%) by the same manner.

(*S*)-1-(3-nitro-[1,1'-biphenyl]-4-yl)but-3-en-2-yl (*R*)-3,3,3-trifluoro-2-methoxy-2-phenylpropanoate

To a stirred solution of (+)-4 (19.6 mg, 72.8 µmol) in dry pyridine-CH_2Cl_2 (1:1, 180 µL) was added DMAP (46.2 mg, 378 µmol) and *S*-α-methoxy-α-(trifluoromethyl)phenylacetyl chloride (MTPA-Cl) (32 µL, 175 µmol). After stirred for 1 h, water was added to quench the reaction. The resulting mixture was extracted with EtOAc (8 mL × 3). The organic layer was washed with brine, dried over Na_2SO_4, and concentrated *in vacuo*. The residual oil was purified by flash chromatography (6–20% EtOAc in hexane) to obtain ***R*-MTPA-(+)-6** (29.1 mg, 82%) as a white solid. ^{1}H NMR (500 MHz, $CDCl_3$): δ_H 3.23 (1H, dd, *J*=14.1 and 8.6 Hz, H-1′), 3.40 (3H, s, -O*CH₃*), 3.48 (1H, dd, *J*=14.1 and 4.6 Hz, H-1′), 5.30 (1H, d, *J*=10.3 Hz, H-4′), 5.39 (1H, d, *J*=17.2 Hz, H-4′), 5.81 (1H, m, H-2′), 5.89 (1H, ddd, *J*=17.2, 10.3 and 6.9 Hz, H-3′), 7.23–7.34 (5H, m, phenyl in MTPA), 7.35 (1H, d, *J*=8.0 Hz, H-3), 7.43 (1H, m, H-10), 7.49 (2H, m, H-9 and 11), 7.60 (2H, m, H-8 and 12), 7.67 (1H, dd, *J*=8.0 and 2.3 Hz, H-4), 8.25 (1H, d, *J*=2.3 Hz, H-6); ^{13}C NMR (126 MHz, $CDCl_3$): δc 37.9, 55.3, 77.2, 84.6 ($J_{C\text{-}F}$=27.8 Hz), 119.1, 123.3 ($J_{C\text{-}F}$=288.5 Hz), 123.4, 126.9, 126.9, 127.4, 127.4, 128.3, 128.3, 128.6, 129.2, 129.2, 129.5, 130.3, 131.3, 131.9, 134.2, 134.3, 138.0, 141.5, 149.5, 165.6; HRMS (*m/z*): $[M+Na]^+$ calcd for $C_{26}H_{22}F_3NO_5Na$ 508.1342; found 508.1309.

R-MTPA-(+)-**6**: $[\alpha]^{25}_D$ +9.2 (MeOH; *c* 0.20).

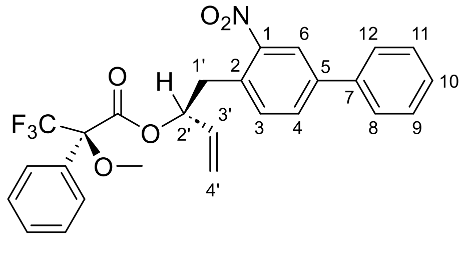

Supplementary Figure S14 Structure of *R*-MTPA-(+)-6

(*R*)-1-(3-nitro-[1,1'-biphenyl]-4-yl)but-3-en-2-yl (*R*)-3,3,3-trifluoro-2-methoxy-2-phenylpropanoate

To a stirred solution of (−)-**4** (16 mg, 59 µmol) in dry pyridine-CH_2Cl_2 (1:1, 135 µL) was added DMAP (38 mg, 310 µmol) and *S*-α-methoxy-α-(trifluoromethyl)phenylacetyl chloride (MTPA-Cl) (26 µL, 140 µmol). After stirred for 1 h, water was added to quench the reaction. The resulting mixture was extracted with EtOAc (8 mL × 3). The organic layer was washed with brine, dried over Na_2SO_4, and concentrated

in vacuo. The residual oil was purified by flash chromatography (6–20% EtOAc in hexane) to obtain ***R*-MTPA-(−)-6** (23.6 mg, 82%) as a white solid. ^{1}H NMR (500 MHz, $CDCl_3$): δ_H 3.19 (1H, dd, *J*=14.3 and 9.2 Hz, H-1′), 3.43 (1H, dd, *J*=14.3 and 4.0 Hz, H-1′), 3.47 (3H, s, -O*CH*$_3$) , 5.35 (1H, d, *J*=10.3 Hz, H-4′), 5.47 (1H, d, *J*=17.2 Hz, H-4′), 5.84 (1H, m, H-2′), 5.95 (1H, ddd, *J*=17.2, 10.3 and 6.9 Hz, H-3′), 7.16 (1H, d, *J*=8.0 Hz, H-3), 7.24–7.28 (3H, m, phenyl in MTPA), 7.36 (2H, m, phenyl in MTPA), 7.43 (1H, m, H-10), 7.49 (2H, m, H-9 and 11), 7.51 (1H, dd, *J*=8.0 and 2.3 Hz, H-4), 7.58 (2H, m, H-8 and 12), 8.19 (1H, d, *J*=2.3 Hz, H-6); ^{13}C NMR (126 MHz, $CDCl_3$): δ_C 37.7, 55.5, 77.0, 84.3 ($J_{C\text{-}F}$=27.8 Hz), 119.8, 123.1 ($J_{C\text{-}F}$=288.5 Hz), 123.2, 126.9, 126.9, 127.1, 127.1, 128.3, 128.3, 128.5, 129.2, 129.2, 129.4, 130.0, 131.2, 132.2, 133.9, 13.3, 138.1, 141.3, 149.4, 165.7; HRMS (*m/z*): [M+Na]$^+$ calcd for $C_{26}H_{22}F_3NO_5Na$ 508.1342; found 508.1348.

R-MTPA-(−)-**7**: $[\alpha]_D^{22}$ −4.6 (MeOH; *c* 0.20).

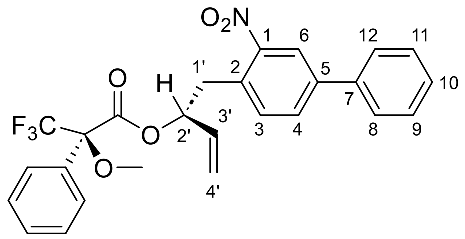

Supplementary Figure S15 Structure of *R*-MTPA-(−)-6

4-(2-bromophenethyl)oxetan-2-one, 2Br-PEO(3)

$AlCl_3$ (7.5 mg, 0.06 mmol) and diisopropylethylamine (DIEA) (39 µL, 0.28 mol) in CH_2Cl_2 (1 mL) was cooled to −25 °C. After being stirred for 5 min at −25 °C, a solution of AgSbF6 (116 mg, 0.34 mmol) in CH_2Cl_2 (1 mL) was added to the stirred mixture. The resulting heterogeneous mixture was added the DIEA (87 µL, 0.62 mmol), acetyl chloride (60 µL, 0.84 mmol) and 3-(2-bromophenyl)propanal (4) (60.3 mg, 0.28 mmol), and then stirred at 0 °C for 6 h. After quenching with 1M HCl, it was extracted with EtOAc (8 mL × 3). The organic layer was washed with brine, dried over Na_2SO_4, and concentrated *in vacuo*. The residual oil was purified by flash chromatography (18% EtOAc in hexane) to obtain 2Br-PEO (36 mg, 50%) as a colorless oil. ^{1}H NMR (500 MHz, $CDCl_3$): δ_H 2.12–2.17 (2H, m), 2.85 (1H, dt, *J*=14.3 and 7.5 Hz), 2.95 (1H, ddd, *J*=14.3, 8.6 and 6.3 Hz), 3.08 (1H, dd, *J*=16.1 and 4.0 Hz), 3.52 (1H, dd, *J*=16.1 and 5.7 Hz), 4.53 (1H, m), 7.10 (1H, ddd, *J*=7.5, 6.9 and 1.7 Hz), 7.23 (dd, *J*=6.9 and 1.7 Hz), 7.26 (1H, ddd, *J*=7.5, 6.9 and 1.1 Hz), 7.55 (1H, dd, *J*=7.5 and 1.1 Hz); ^{13}C NMR (126 MHz, $CDCl_3$): δ_C 31.7, 34.7, 43.0, 70.3, 124.3, 127.7, 128.3, 130.5, 133.1, 139.4, 168.0; HRMS (*m/z*): [M+Na]$^+$ calcd for $C_{11}H_{11}BrO_2Na$ 276.9835; found 276.9879.

dYLG assay

To produce KAI2 protein, KAI2 cDNA was cloned into the pCold II vector, and KAI2 protein was expressed and purified as previously described(5). Briefly, the plasmid was transformed into BL21, the resulting transformant cells were precultured overnight in 2.5 mL LB medium and then cells were grown in 200 mL TB medium at 35 °C to an OD_{600} ~0.6. Protein expression was induced by IPTG addition, and inductions were conducted at 16 °C for 20 h. Cells were harvested and then suspended in the protein extraction buffer (50 mM Tris-HCl pH 8.0, BugBuster protein extraction reagent, and 20U of Benzonase, and the constituent proteins were purified by affinity column chromatography. The purified KAI2 (1 μg) was incubated with dYLG (0.5 μM) in the absence or presence of dMGer analogs for 10 min. Fluorescence intensity was measured using the Varioskan LUX plate reader (Thermo Fisher Scientific), with excitation and detection wavelengths of 480 and 520 nm, respectively. The IC_{50} values were calculated using the Enzyme Kinetics module of the SigmaPlot 14 program.

For the inhibitory activity persistence test, fluorescence intensity was recorded with excitation and detection wavelengths of 480 and 520 nm, respectively, over 60 min at 1−min intervals.

Enzymatic degradation of (+)-/(−)-dMGer analogs and 2Br-PEO by purified KAI2 protein

A reaction mixture containing purified KAI2 (3 μM) and (+)-dMGer analogs or 2Br-PEO (10 μM final concentration) in PBS (pH 7.6) was incubated at 30 °C for 10 min. After adding 4-phenylphenol (0.3 nmol) as the internal standard, the residual (+)-dMGer analogs or 2Br-PEO were extracted with EtOAc (3×150 μL) and concentrated in *vacuo*. The dried sample was then dissolved in 50 μL of MeOH, after which a 10 μL aliquot was analyzed by HPLC. The HPLC conditions were as follows: column, Kinetex PS C_{18} (100 × 4.6 mm, 2.6 μm, Shimadzu GLC Ltd., Tokyo, Japan); solvent, 60% MeOH in H_2O; flow rate, 1.5 mL min^{-1}; and detection wavelength, 190−350 nm. Data are presented as the averages from three independent experiments.

Enzymatic assay with (+)-dMGer and (+)-1′-carba-dMGer

The enzyme assay was determined by measuring the release of 4-hydroxy-3-nitrobipheny after hydrolysis of (+)-dMGer and (+)-1′-carba-dMGer by KAI2 protein. A reaction mixture containing purified KAI2 (0.63 μM) and (+)-dMGer or (+)-1′-carba-dMGer (2–128 μM) in PBS (pH 7.6) was incubated at 30 °C for 10 min. After adding 4-phenylphenol (0.3 nmol) as the internal standard, the enzymatic products were extracted with EtOAc (3×150 μL) and concentrated in vacuo. The dried sample was then dissolved in 50 μL of MeOH, after which a 10 μL aliquot was analyzed by HPLC. The HPLC conditions were as described above. Enzyme activity was confirmed by determining the amount of 4-hydroxy-3-nitrobipheny in control experiments before each set of measurements. K_m, V_{max} and k_{cat} were determined using the Enzyme Kinetics module of SigmaPlot 14 software.

For determination of k_{cat} as the rate constant of the pre-steady-state phase(6), a reaction mixture containing purified KAI2 (3 μM) and (+)-dMGer or (+)-1′ -carba-dMGer (0.3–100 μM) in PBS (pH

7.6) was incubated at 30 °C for 10 min. In this equation $V_{max} = k_{cat} \times [E_{total}]$. k_{cat} is equivalent to k_2 and $K_{1/2}$ is equivalent to k_{-1}/k_1 in the equation in *SI Appendix* Fig. 12.

Protein preparation and crystallization

The tobacco etch virus (TEV) protease recognition site was inserted between the His_6-tag and KAI2 sequences on the pCold II vector for tag removal. The plasmid was then transformed into BL21 (DE3), the resulting transformant cells were incubated at 37 °C to an OD_{600} ~0.6. Protein expression was induced by IPTG addition and incubation at 15 °C for 23 h, after which cells were harvested, frozen, thawed and sonicated. Protein was then purified from this preparation using a Ni-Sepharose resin (GE Healthcare Bio-Sciences AB) and eluted with binding buffer (50 mM Tris-HCl, pH 8.0) supplemented with 250 mM imidazole. The N-terminal His_6 tag was removed by treatment with TEV protease overnight. TEV protease and uncleaved His_6-KAI2 were removed using the Ni-Sepharose resin. KAI2 protein was further purified by size exclusion chromatography on a Superdex 75 increase 10/300 column (Cytiva), dialyzed into crystallization buffer [10 mM PIPES-Na (pH 6.5)] and concentrated to 11 mg mL^{-1} using a Vivaspin 20 (Sartorius).

Crystallization was performed using the sitting-drop vapor-diffusion method at 20 °C, and crystallization buffer comprised 0.1 M Tris-HCl (pH 7.0 to 9.0) with 1.25 to 2 M ammonium sulfate and 12% (v/v) glycerol as the reservoir buffer. Drops of protein solution (1.0 μL, 11 mg mL^{-1}) were mixed with 1.0 μL of reservoir buffer.

Data collection, structure determination and refinement

A crystal of KAI2 was dipped in the reservoir with 30% glycerol as a cryoprotectant and mounted on the goniometer. Diffraction of the 1.12 Å X-ray by the crystal was measured 360° with oscillation range 1° under 100 K. To solve the ligand complex structure, a crystal of KAI2 was soaked in the reservoir containing 100 μM (+)-6′-carba-dMGer for 2 h at 20 °C. The soaked crystal was dipped in the reservoir with 30% glycerol as a cryoprotectant. Diffraction of the 0.72 Å X-ray by the crystal was measured 360° with oscillation range 0.25° under 100 K. Images were processed by XDS (10.1107/S0907444909047337) and the phase was determined by molecular replacement using Phaser (10.1107/S0021889807021206) and 4JYP as a template. Models were improved using Coot (10.1107/S0907444904019158) and refined by Phenix refine (10.1107/S2059798319011471).

Mass spectrometric analysis of covalent modification with in-gel digestion

To detect covalent modification of the KAI2 protein, the trypsin- and chymotrypsin-digested peptides of KAI2 were analyzed by LC-MS/MS. KAI2 protein (20 μM) was incubated in 100 mM HEPES (pH 7.4) in the presence or absence of (+)-dMGer analogs (500 μM) for 30 min at room temperature. The reaction mixture was denatured and subjected to SDS-PAGE. The gel bands of KAI2 were excised, reduced with

10 mM DTT, and alkylated with 55 mM iodoacetamide. Then, the in-gel digestion was performed with trypsin (Promega) and chymotrypsin (Promega) in 50 mM NH_4CO_3 aqueous solution at 37 °C overnight. The digested peptides were extracted twice with 3% (v/v) formic acid in 50% (v/v) acetonitrile aqueous solution for 20 min. After centrifugation, 50 μL of the supernatant was collected and concentrated, and then 10 μL of 0.3% formic acid solution was added to prepare the LC-MS/MS sample. LC-MS/MS analysis was performed using a linear ion trap time-of-flight mass spectrometer, NanoFrontier eLD (Hitachi High-Tech). The digested peptides were separated using a NanoFrontier nLC (Hitachi High-Tech) equipped with a Capillary EX-Nano column (0.05 × 150 mm, GL Sciences). Separation and elution of the peptide were performed with a linear gradient from 5% to 40% of solvent A (2% acetonitrile and 0.1% formic acid) and solvent B (98% acetonitrile and 0.1% formic acid) for 60 min at a flow rate of 200 nL/min. The eluted peptides were loaded into a nano-electrospray ionization source using an uncoated SilicaTip (New Objective), and MS and MS/MS spectra were acquired in a positive ion mode at a scan mass range of m/z 100–2000. Covalent modification of the peptides was detected using a software platform for proteomics, PEAKS ver. 7.0(7).

^{1}H NMR spectrum of Compound 3

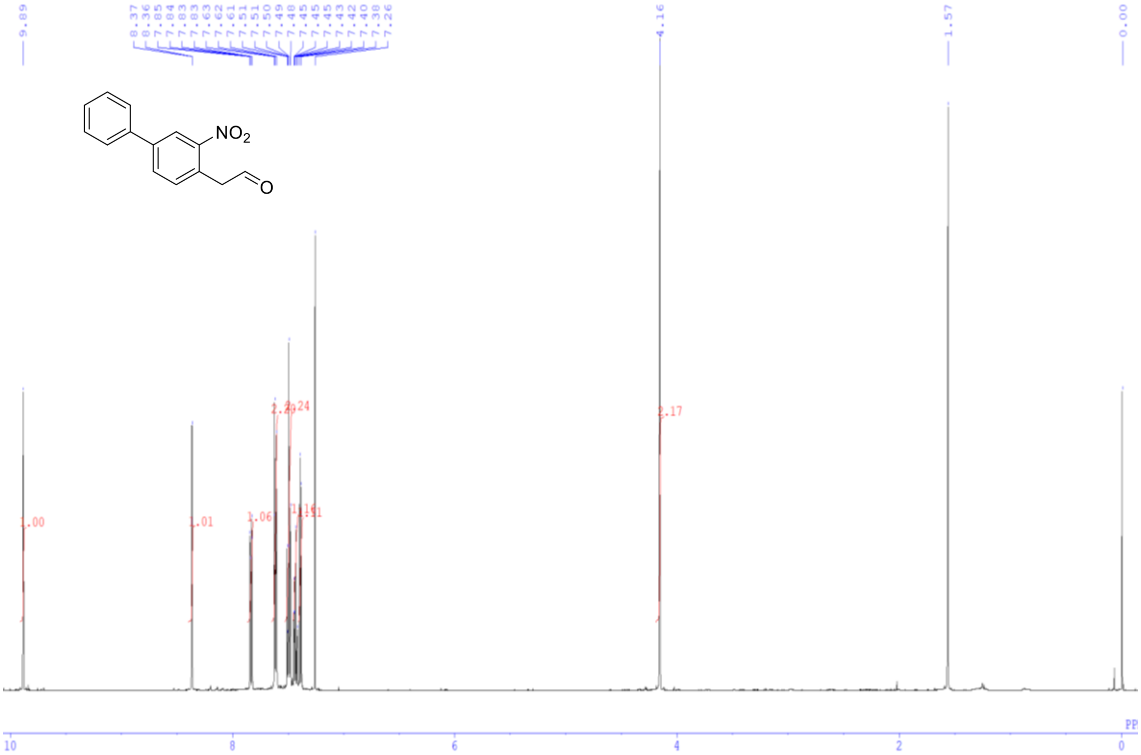

^{13}C NMR spectrum of Compound 3

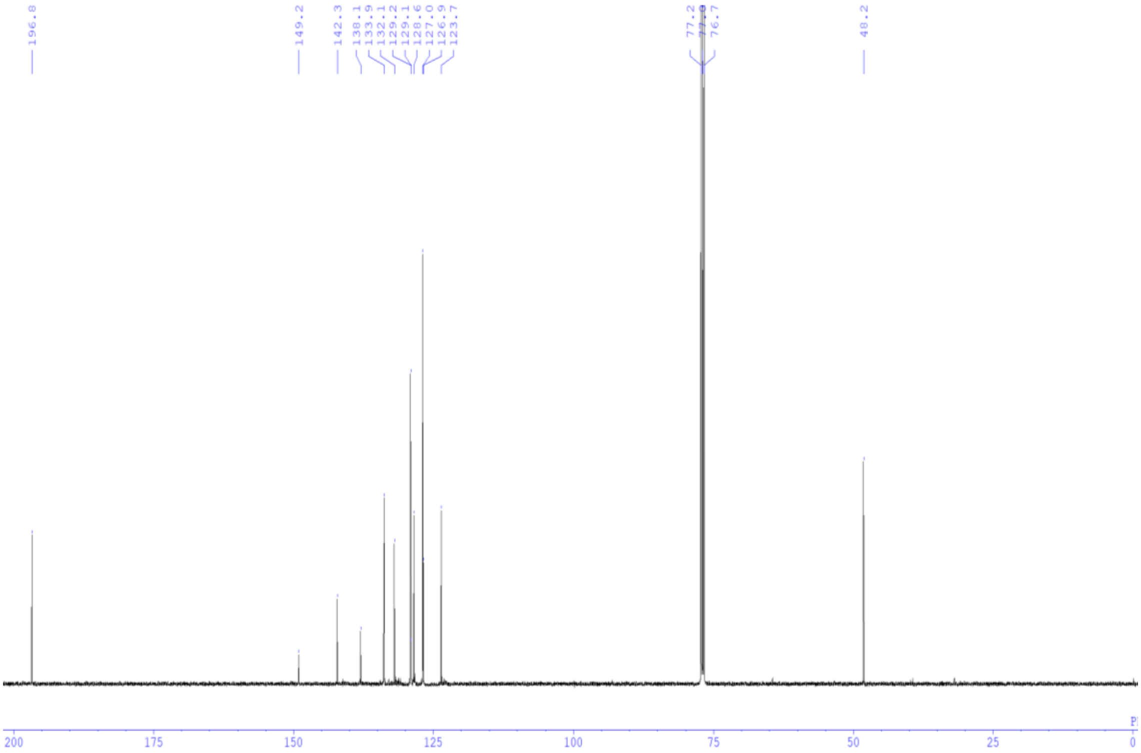

Supplementary Figure S16 ^{1}H and ^{13}C NMR Spectrums of Compound 3.

^{1}H NMR spectrum of Compound 4

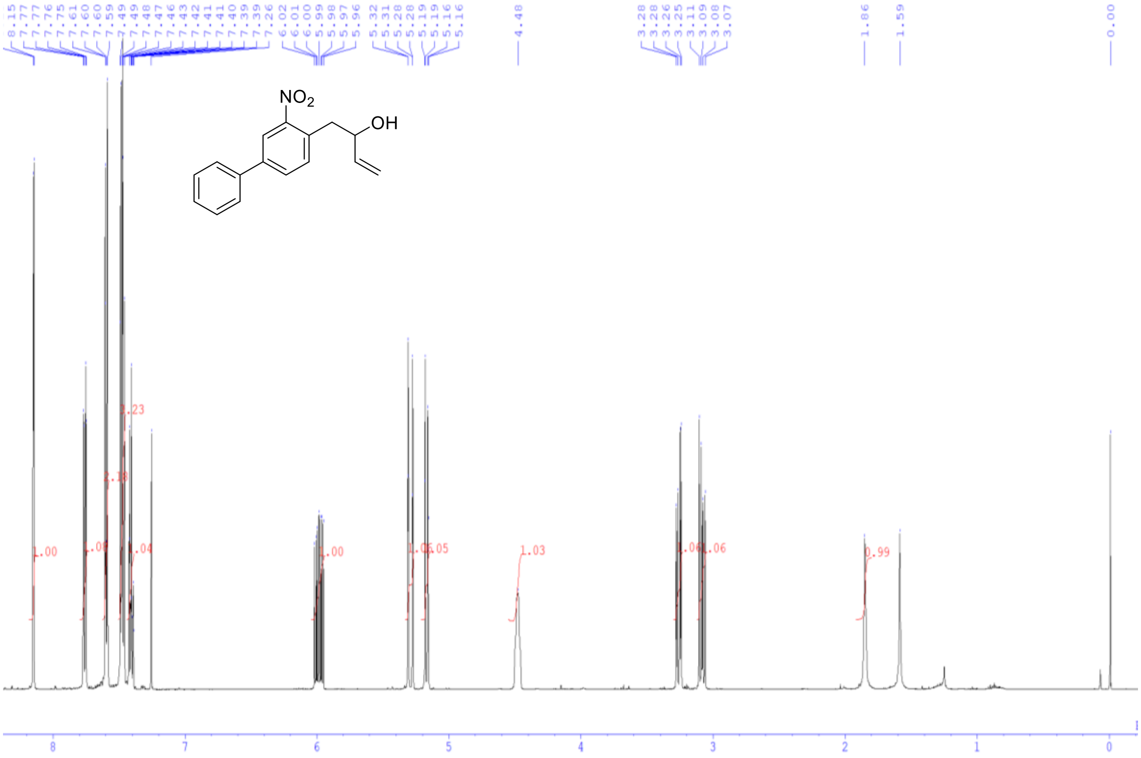

^{13}C NMR spectrum of Compound 4

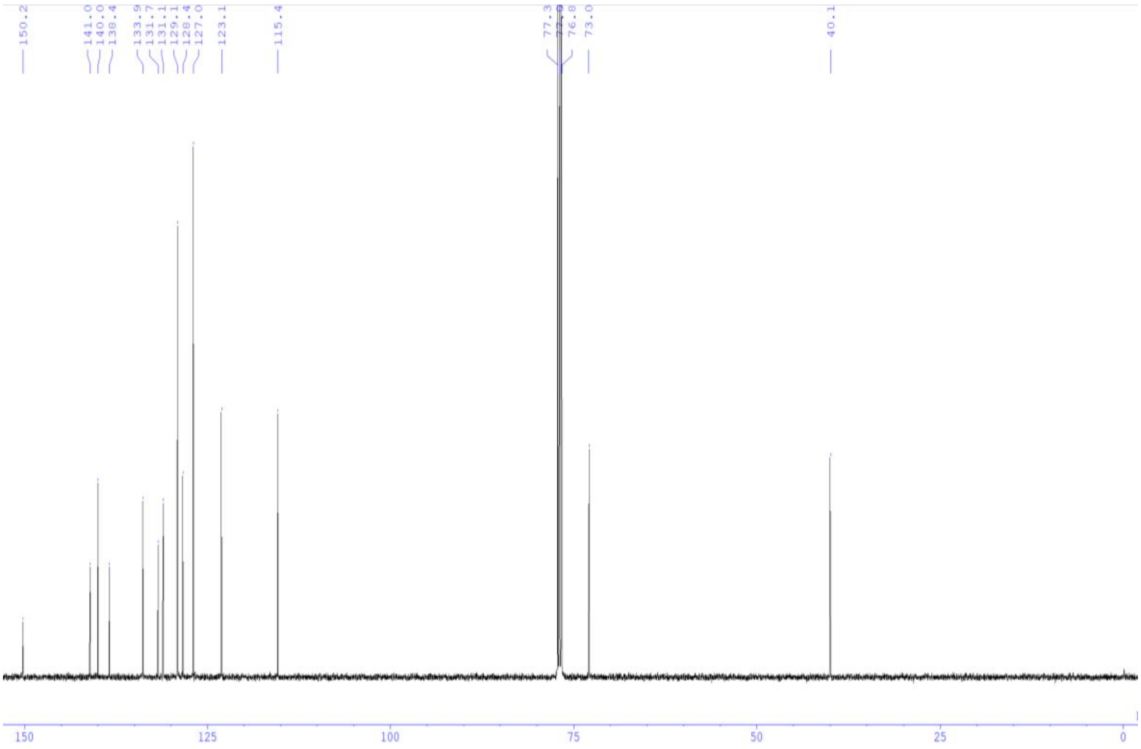

Supplementary Figure S17 ^{1}H and ^{13}C NMR Spectrums of Compound 4.

^{1}H NMR spectrum of Compound 5

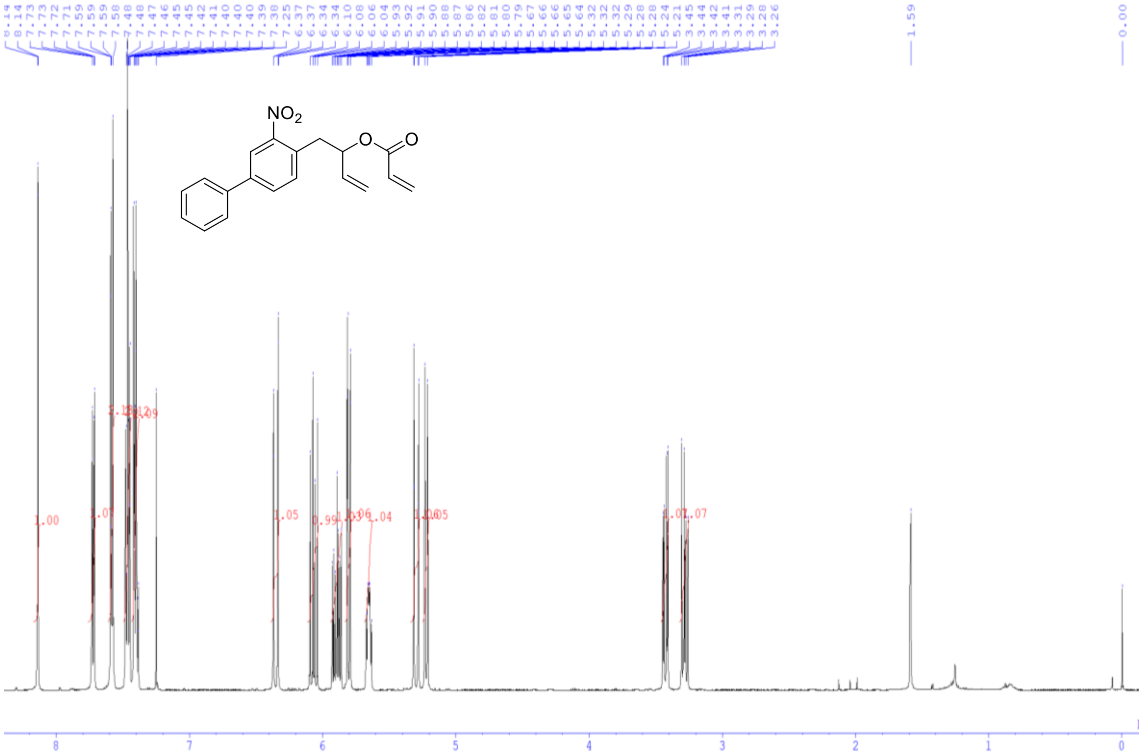

^{13}C NMR spectrum of Compound 5

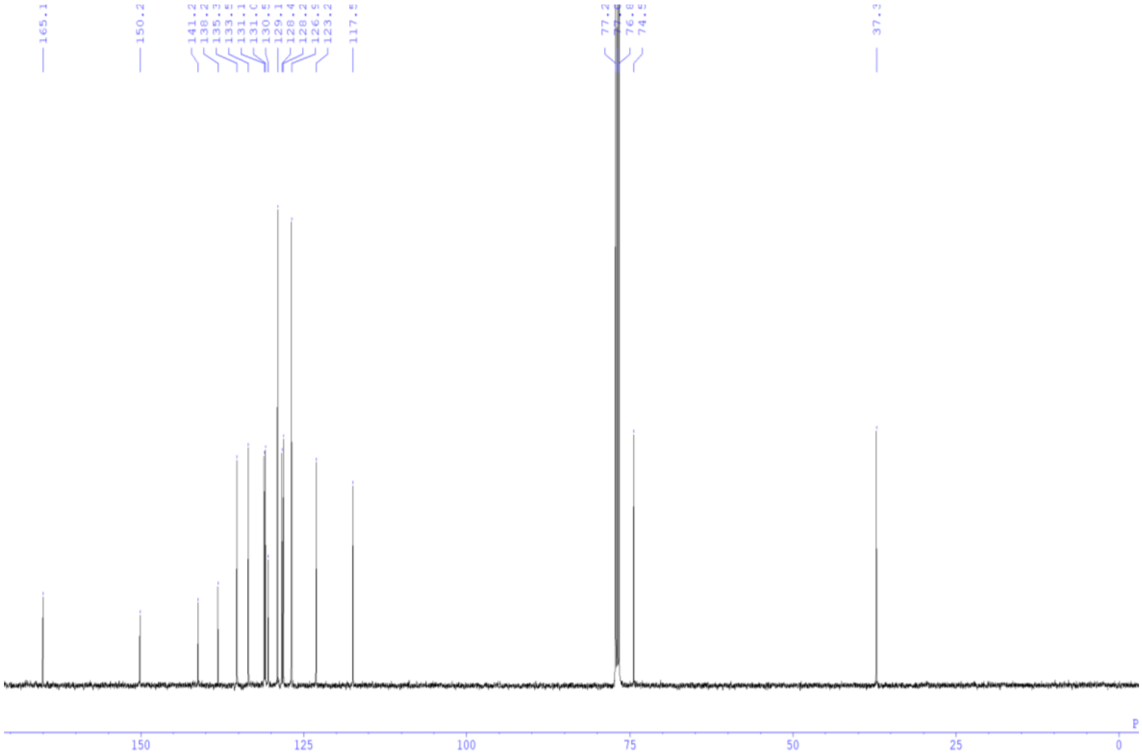

Supplementary Figure S18 ^{1}H and ^{13}C NMR Spectrums of Compound 5.

^{1}H NMR spectrum of (±)-6′-carba-dMGer

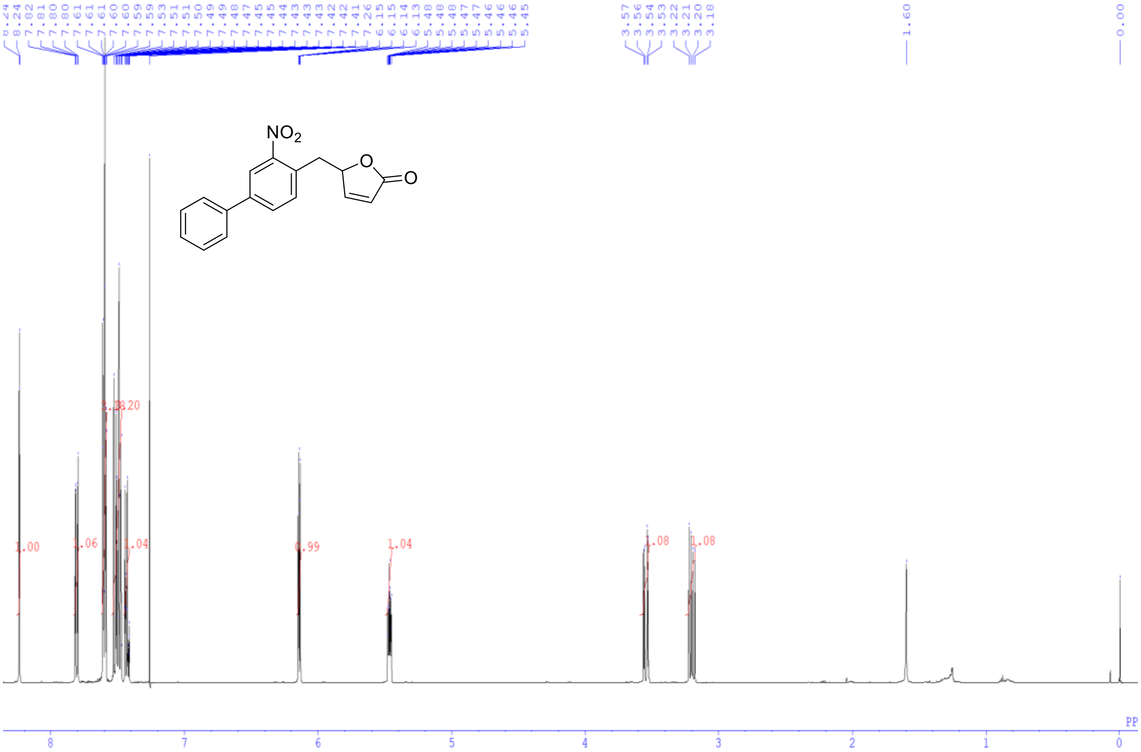

^{13}C NMR spectrum of (±)-6′-carba-dMGer

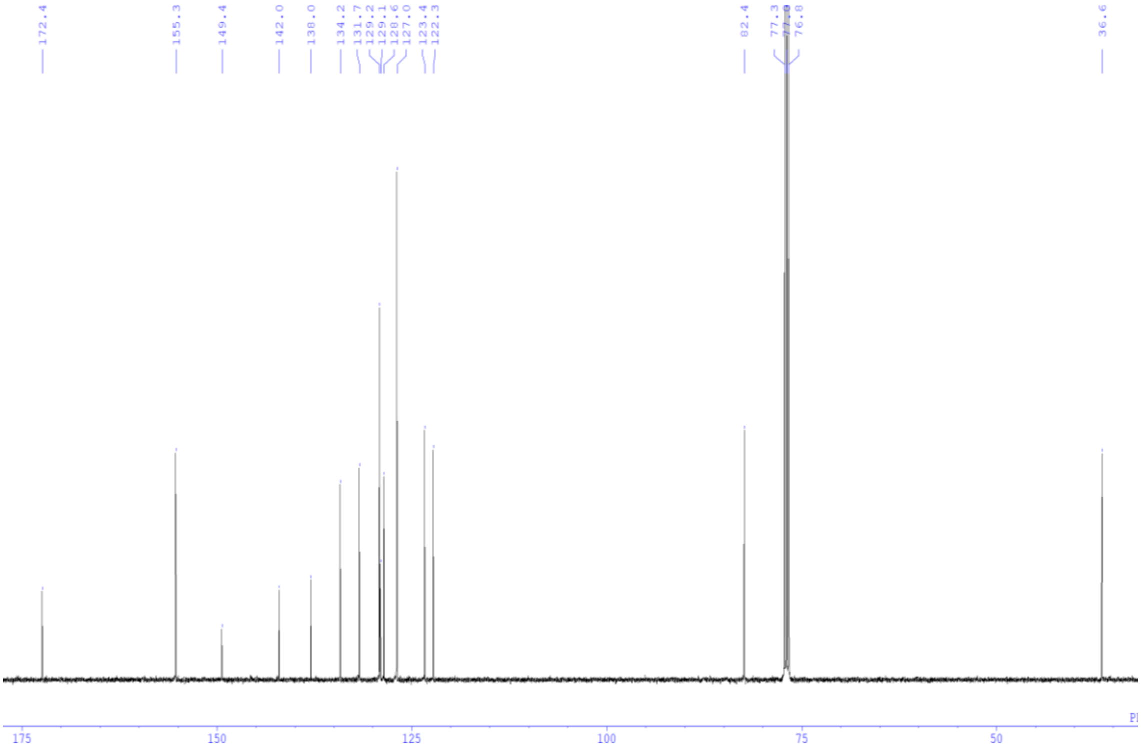

Supplementary Figure S19 ^{1}H and ^{13}C NMR Spectrums of (±)-6′-carba-dMGer.

^{1}H NMR spectrum of (±)-1′-carba-dMGer

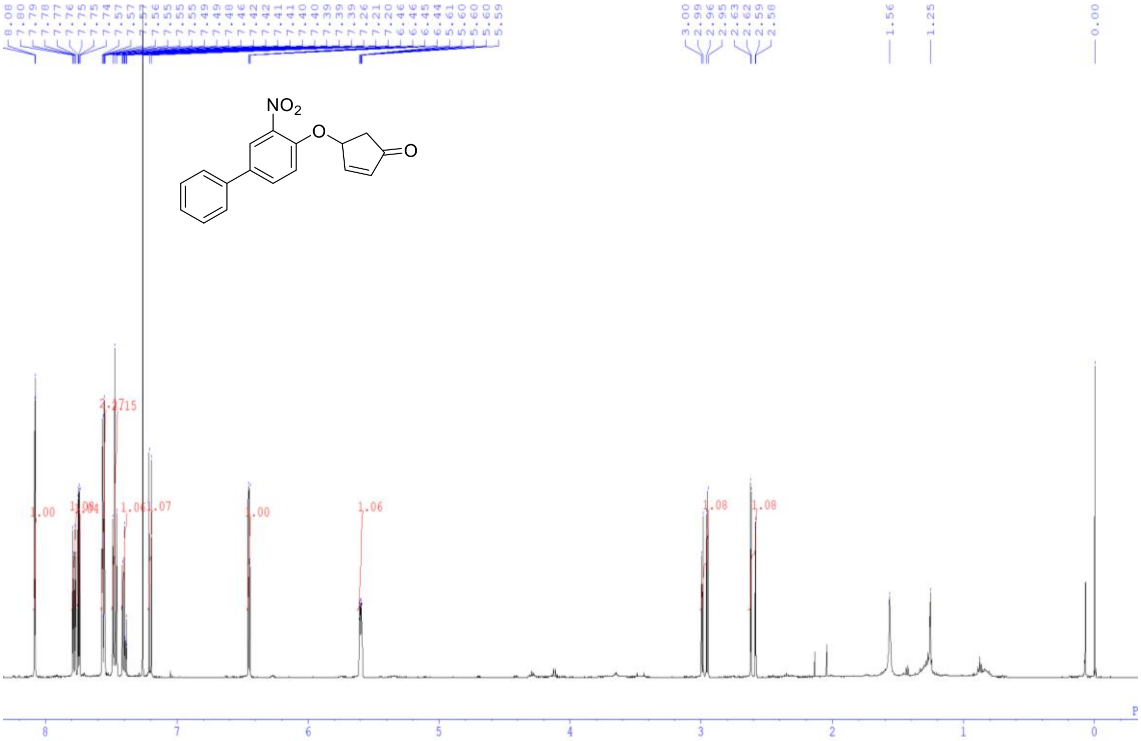

^{13}C NMR spectrum of (±)-1′-carba-dMGer

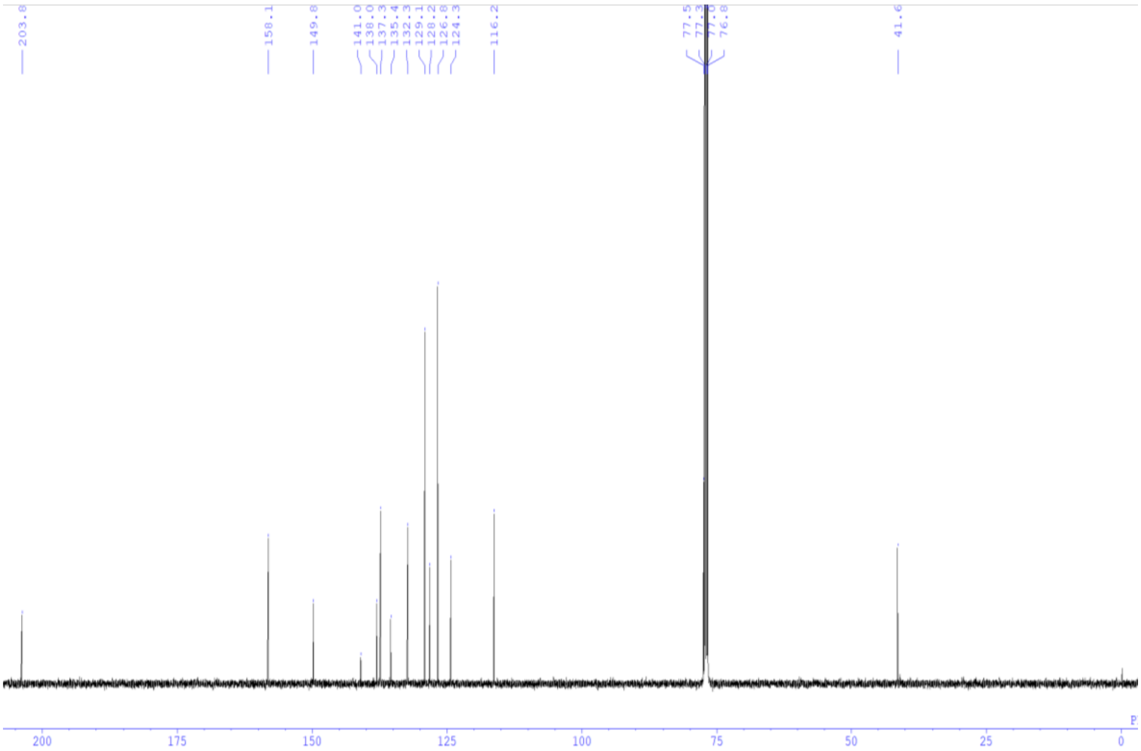

Supplementary Figure S20 ^{1}H and ^{13}C NMR Spectrums of (±)-1′-carba-dMGer.

^{1}H NMR spectrum of Compound *S*-(+)-6

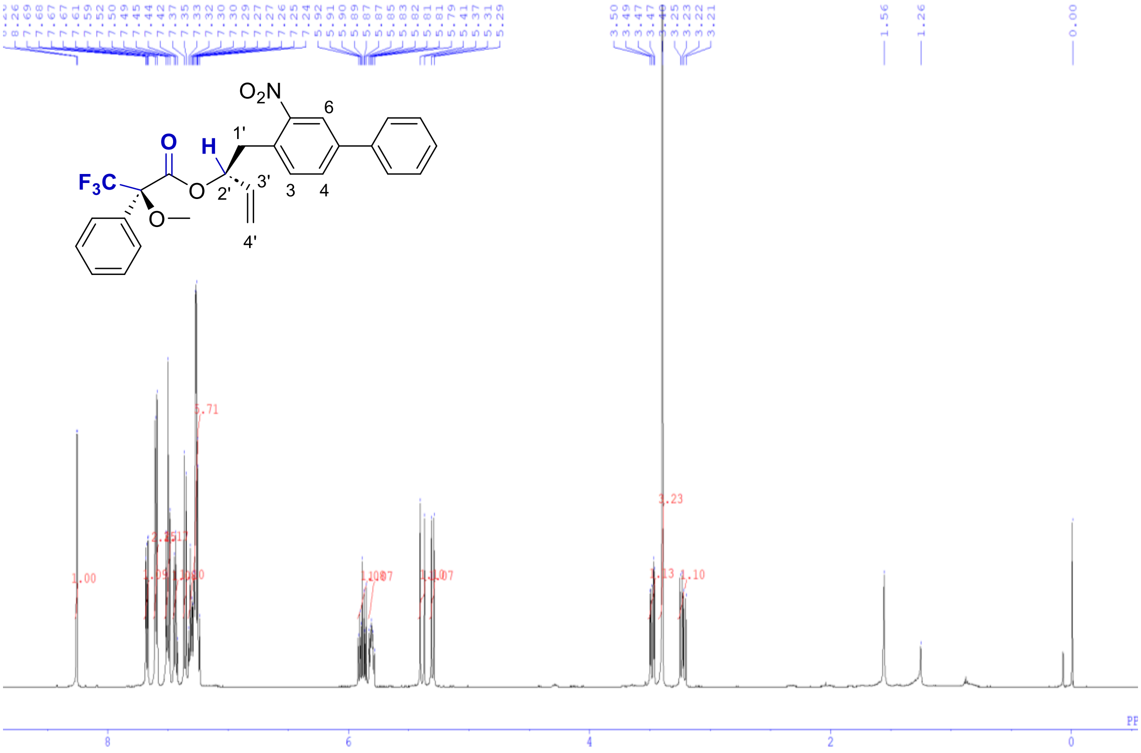

^{13}C NMR spectrum of Compound *S*-(+)-6

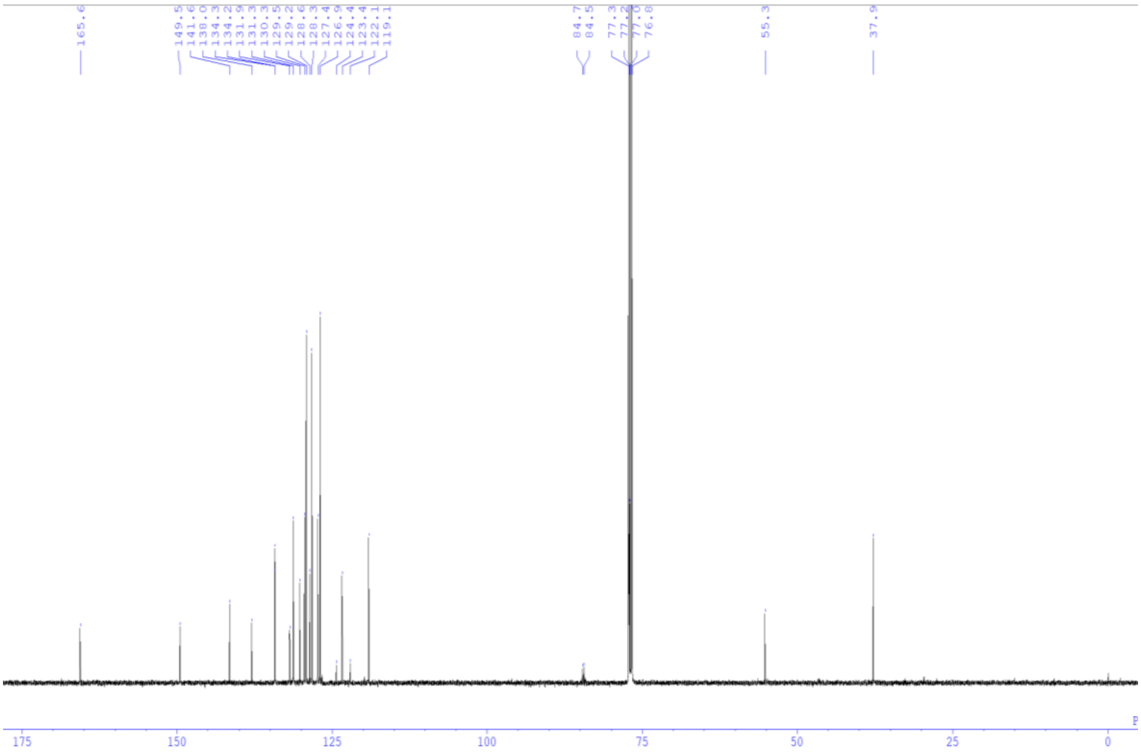

Supplementary Figure S21 ^{1}H and ^{13}C NMR Spectrums of Compound *S*-(+)-6.

^{1}H NMR spectrum of Compound *R*-(−)-6

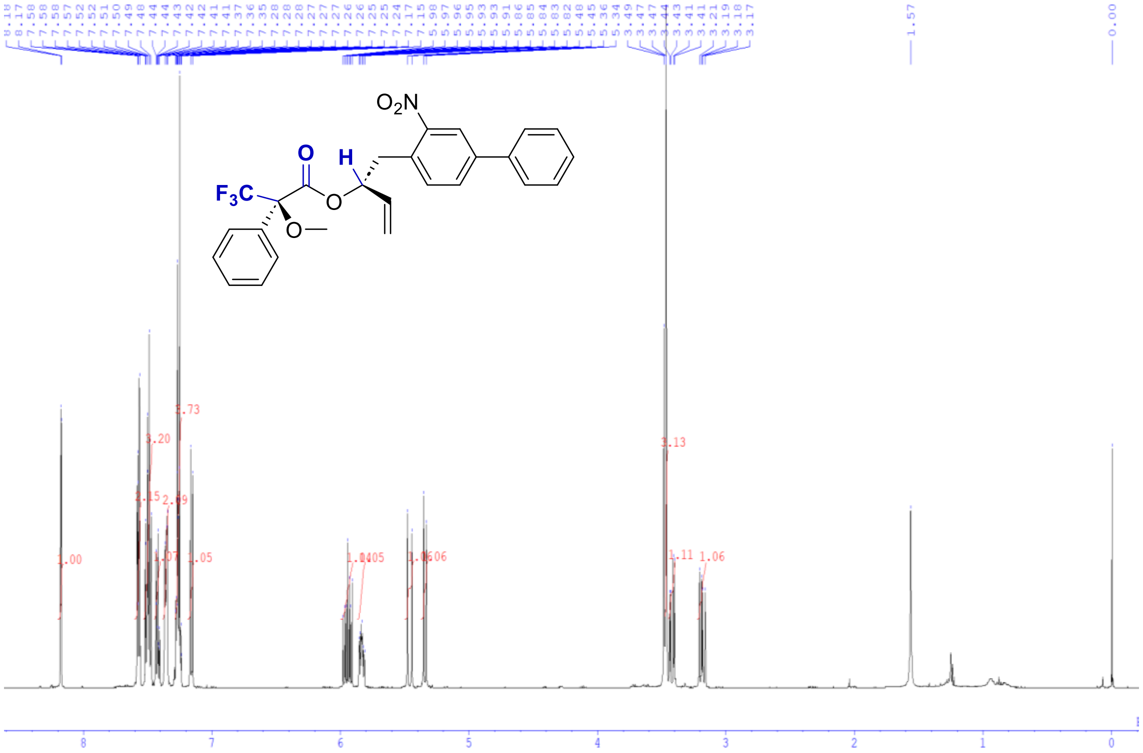

^{13}C NMR spectrum of Compound *R*-(−)-6

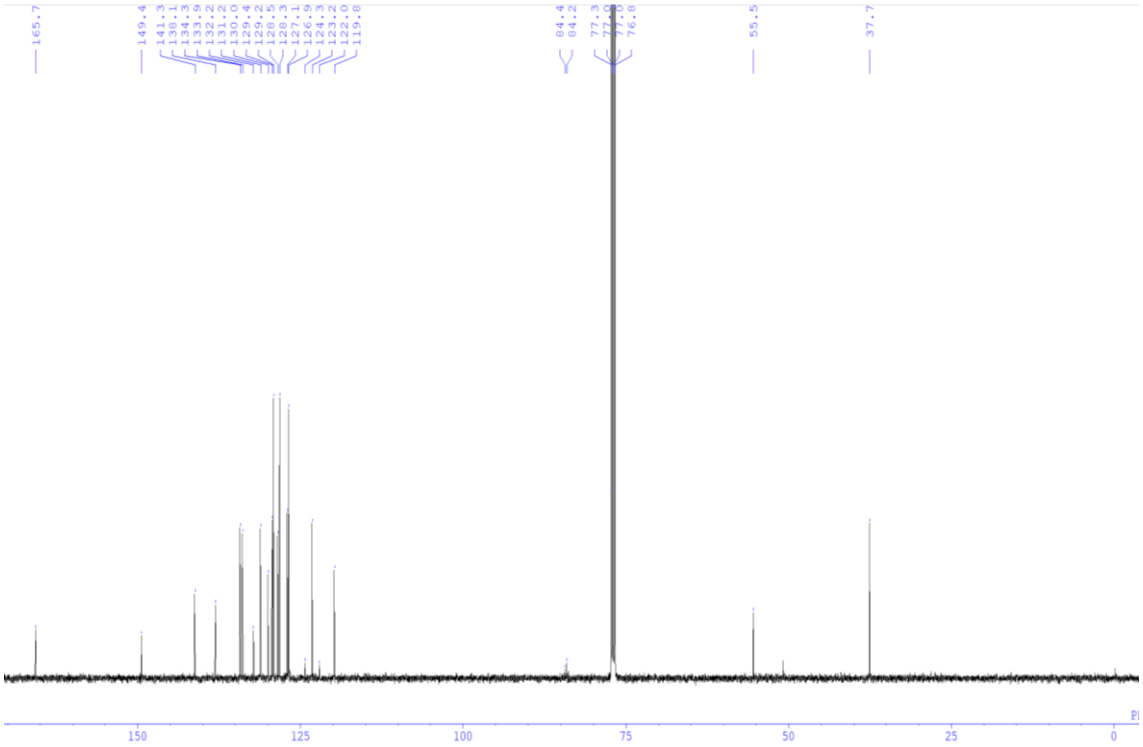

Supplementary Figure S22 ^{1}H and ^{13}C NMR Spectrums of Compound *R*-(−)-6.

^{1}H NMR spectrum of 2Br-PEO

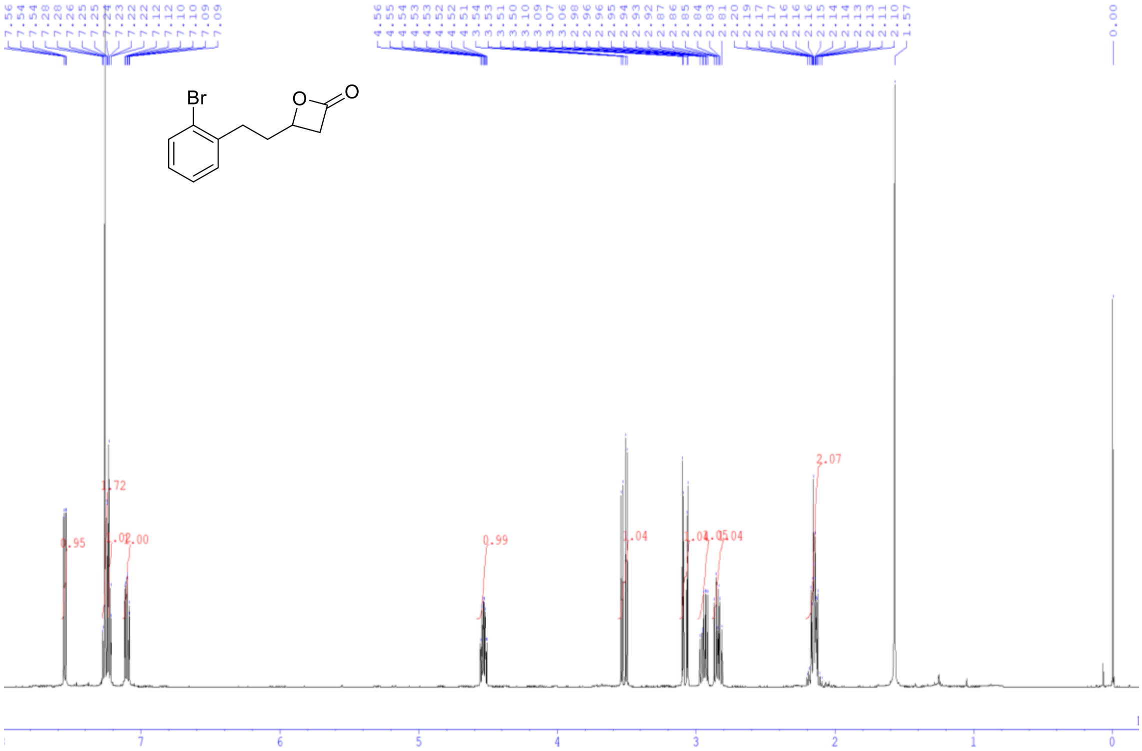

^{13}C NMR spectrum of 2Br-PEO

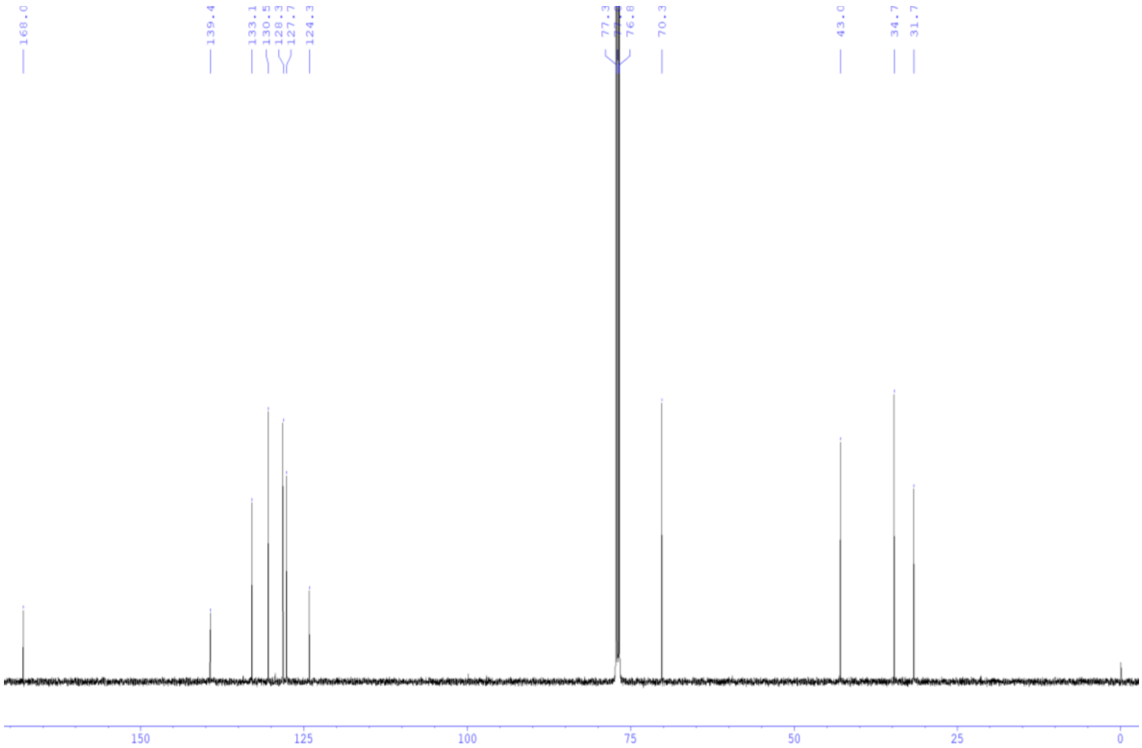

Supplementary Figure S23 ^{1}H and ^{13}C NMR Spectrums of 2Br-PEO.

References

1. P. K. Suryadevara, *et al.*, Structurally Simple Inhibitors of Lanosterol 14α-Demethylase Are Efficacious In a Rodent Model of Acute Chagas Disease. *J. Med. Chem.* **52**, 3703–3715 (2009).

2. M. Bollenbach, *et al.*, Phenylpyridine-2-ylguanidines and rigid mimetics as novel inhibitors of TNFα overproduction: Beneficial action in models of neuropathic pain and of acute lung inflammation. *Eur. J. Med. Chem.* **147**, 163–182 (2018).

3. S. G. Nelson, Z. Wan, T. J. Peelen, K. L. Spencer, Catalyzed acyl halide□aldehyde cyclocondensations. New insights into the design of catalytic cross aldol reactions. *Tetrahedron Lett.* **40**, 6535–6539 (1999).

4. E. M. Rochette, W. Lewis, A. G. Dossetter, R. A. Stockman, Highly diastereoselective radical cyclisations of chiral sulfinimines. *Chem. Commun.* **49**, 9395–9397 (2013).

5. K. Fukui, K. Arai, H. Kasahara, T. Asami, K. Hayashi, Synthetic agonist of HTL/KAI2 shows potent stimulating activity for *Arabidopsis* seed germination. *Bioorg. Med. Chem. Lett.* **29**, 2487–2492 (2019).

6. A. De Saint Germain, *et al.*, An histidine covalent receptor and butenolide complex mediates strigolactone perception. *Nat. Chem. Biol.* **12**, 787–794 (2016).

7. B. Ma, *et al.*, PEAKS: powerful software for peptide de novo sequencing by tandem mass spectrometry. *Rapid Commun. Mass Spectrom.* **17**, 2337–2342 (2003).
